# Birthweight and risk of chronic kidney disease after a type 2 diabetes diagnosis in the DD2 cohort

**DOI:** 10.1007/s00125-024-06357-4

**Published:** 2025-02-01

**Authors:** Aleksander L. Hansen, Christian F. Christiansen, Charlotte Brøns, Leonie M. Engelhard, Torben Hansen, Jens S. Nielsen, Peter Vestergaard, Kurt Højlund, Niels Jessen, Michael H. Olsen, Henrik T. Sørensen, Peter Rossing, Reimar W. Thomsen, Allan Vaag

**Affiliations:** 1 Steno Diabetes Center Copenhagen, Herlev, Denmark; 2 Department of Clinical Epidemiology, Aarhus University Hospital, and Department of Clinical Medicine, Aarhus University, Aarhus, Denmark; 3 Lund University Diabetes Center, Lund University, Lund, Sweden; 4 Novo Nordisk Foundation Center for Basic Metabolic Research, University of Copenhagen, Copenhagen, Denmark; 5 Steno Diabetes Center Odense, Odense University Hospital, Odense, Denmark; 6 Steno Diabetes Center North Denmark, Aalborg University Hospital, Aalborg, Denmark; 7 Steno Diabetes Center, Aarhus University Hospital, Aarhus, Denmark; 8 Department of Clinical Medicine, University of Copenhagen, Copenhagen, Denmark; 9 Department of Internal Medicine and Steno Diabetes Center Zealand, Holbæk Hospital, Holbæk, Denmark; 10 Department of Endocrinology, Skåne University Hospital, Malmö, Sweden

**Keywords:** Birthweight, Chronic kidney disease, Cohort study, Epidemiology, Fetal programming, Type 2 diabetes

## Abstract

**Aims/hypothesis:**

Low birthweight (LBW) is associated with younger age, less obesity and more hypertension among people recently diagnosed with type 2 diabetes, as well as increased cardiovascular morbidity and mortality risk. It is not known whether LBW is associated with an increased risk of incident chronic kidney disease (CKD) among people with a type 2 diabetes diagnosis.

**Methods:**

Original midwife records were retrieved for 5982 participants with recently diagnosed type 2 diabetes enrolled in the Danish Center for Strategic Research in Type 2 Diabetes (DD2) cohort between 2010 and 2024. They were followed until first incident CKD diagnosis, defined as either two eGFR measurements <60 ml/min per 1.73m^2^ or two urine albumin/creatinine ratio (UACR) measurements >3 mg/mmol, each 90–365 days apart. Confounder-standardised 10 year risks of CKD were estimated, with death considered as a competing risk. Adjusted hazard ratios (aHRs) for CKD were computed using Cox and spline regression analyses. All analyses were controlled for differences in sex, age at enrolment, calendar year at birth, family history of diabetes and born-at-term status. Mixed-effects models were used to examine the trajectories of eGFR and UACR following enrolment.

**Results:**

A total of 1501 incident CKD endpoints occurred, corresponding to an incidence rate of 42.4 per 1000 person-years over a median follow-up time of 8.3 years. Spline models with birthweight as a continuous measure showed progressively increasing aHRs for CKD with decreasing birthweight. The 10-year standardised risk of CKD was 36.0% in people with LBW (<2500 g) and 30.6% in people with a normal birthweight (2500–4000 g), yielding a risk difference (RD) of 5.5% (95% CI −0.5%, 11.8%) and an aHR of 1.23 (95% CI 0.98, 1.55). People with type 2 diabetes and high birthweight (>4000 g) had a similar 10-year standardised CKD risk compared with normal birthweight (33.1% and 30.6%, respectively). This yielded an RD of 2.5% (95% CI −1.6%, 6.7%) and an aHR of 1.10 (95% CI 0.93, 1.29). In mixed-effects models examining eGFR and UACR trajectories, each 1 kg decrease in birthweight was associated with a 6.6% (95% CI 1.9, 11.1) increase in UACR, whereas no association was found for eGFR.

**Conclusions/interpretation:**

A history of LBW was associated with elevated risk of CKD among people with a recent type 2 diabetes diagnosis, although the precision of risk estimates was limited.

**Graphical Abstract:**

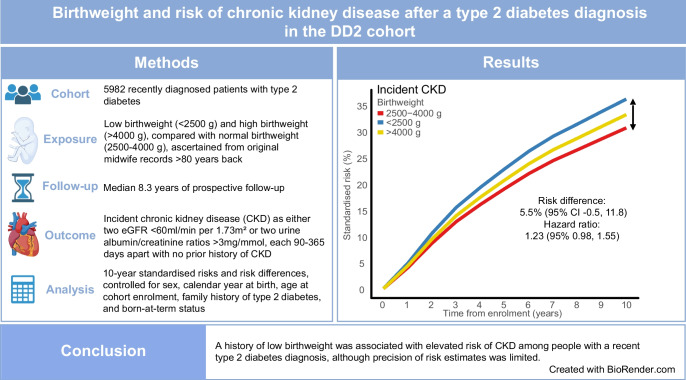

**Supplementary Information:**

The online version contains peer-reviewed but unedited supplementary material available at 10.1007/s00125-024-06357-4.


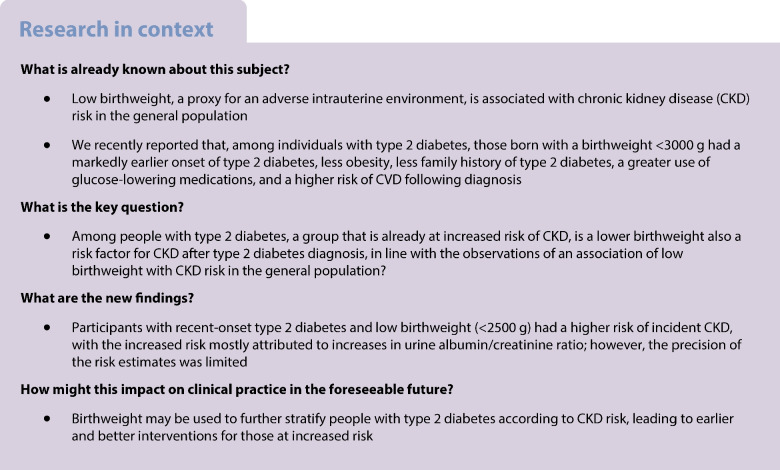


## Introduction

Adverse conditions during fetal development can have long-term effects on the developing organism, predisposing to diseases in adulthood [1]. Low birthweight (LBW), a proxy for an adverse intrauterine environment, is associated with elevated risks of hypertension, type 2 diabetes, CVD and chronic kidney disease (CKD) [1, 2]. In people with insulin-dependent diabetes, LBW has been shown to be associated with reduced nephron numbers, glomerular volume and glomerular filtration rates, and faster progression of kidney disease [2, 3]. Diabetes is the leading global risk factor for CKD [4, 5], which in turn exacerbates the risk of many other diabetes complications, including CVD, infections, adverse drug reactions, reduced quality of life, and premature mortality [6]. We recently reported that individuals with type 2 diabetes and LBW had markedly earlier type 2 diabetes onset, less obesity and less family history of diabetes, but more comorbidities, including hypertension and a greater use of glucose-lowering medications, alongside an elevated risk of CVD [7, 8]. Accordingly, some type 2 diabetes complications, including CKD, may originate from the same adverse fetal environment that is associated with type 2 diabetes development, rather than occurring only as consequence of hyperglycaemia or other secondary dysmetabolic changes. Research on the risk of incident CKD in people born with LBW is scarce and has focused on the general population [2], subgroups of insulin-treated diabetes patients [9, 10] or smaller cross-sectional studies among Pima Indians [11]. Larger studies specifically examining individuals with newly diagnosed type 2 diabetes together with valid birth data are needed to assess the risk of subsequent diagnosis with incident CKD. Using a prospective cohort design, we therefore aimed to examine whether LBW is associated with elevated risk of CKD development and progression following a type 2 diabetes diagnosis, using a large cohort of newly diagnosed people with type 2 diabetes.

## Methods

### Study design and study population

The Danish Center for Strategic Research in Type 2 Diabetes cohort (DD2 cohort) is a nationwide cohort of individuals who were recently diagnosed with type 2 diabetes, starting in November 2010. Participants are recruited at general practitioners and hospital specialist outpatient clinics [12]. The cohort’s design, enrolment, implementation, biobank and characteristics have been described previously [13]. In brief, clinicians identify eligible people who are newly diagnosed with type 2 diabetes and complete an online questionnaire, including physical examination. Urine and fasting blood samples are collected and stored in a biobank [12]. The unique civil registration number assigned to all Danish citizens links the DD2 cohort to extensive Danish health and administrative registries [14]. Detailed information on collected variables is available in previous publications [13]. Information on baseline covariates, definitions and codes is provided in electronic supplementary material [ESM] Tables 1 and 2.

### Intrauterine environment variables

The feasibility of accessing birth data for Danish residents born in Denmark using the Danish National Archives and the Danish Medical Birth Registry has been established [7, 15]. Such data included information on birthweight, non-singleton birth status and born-at-term status [7]. Based on our recent findings [7], we analysed birthweight using three categorisations: (1) standard clinical definitions of LBW (<2500 g) and high birthweight (>4000 g), using normal birthweight (2500–4000 g) as the reference [16]; (2) categories defined as below the lowest quartile (<25%; <3000 g) and above the highest quartile (>75%; >3700 g), using those between the middle quartiles (25–75%; 3000–3700 g) as reference; and (3) birthweight as a continuous variable.

### Outcome variables

Outcome variables were obtained from the National Laboratory Database, which contains information on all laboratory measurements from all major biochemical and immunological laboratories in Denmark, with truncated coverage initially and complete nationwide coverage starting in 2015 [17, 18]. Based on the Kidney Disease: Improving Global Outcomes (KDIGO) criteria, incident CKD was defined as: (1) two eGFR measurements <60 ml/min per 1.73m^2^ taken ≥90 days to ≤365 days apart; or (2) two urine albumin/creatinine ratio (UACR) measurements >3 mg/mmol taken ≥90 days to ≤365 days apart, without a history of CKD at enrolment [19, 20]. CKD progression [19] was defined as: two eGFR measurements <60 ml/min per 1.73m^2^ taken ≥90 to ≤365 days apart, with a ≥25% drop from the first measurement, concurrent with a drop in GFR category to G3a (eGFR 45–59 ml/min per 1.73m^2^), G3b (eGFR 30–44 ml/min per 1.73m^2^), G4 (eGFR 15–29 ml/min per 1.73m^2^) or G5 (eGFR <15 ml/min per 1.73m^2^), without a history of CKD at enrolment [21]. In an additional analysis, we combined the CKD progression outcome with UACR measures, including eGFR definitions or two UACR measurements >3 mg/mmol taken ≥90 days to ≤365 days apart with a ≥25% increase from the first UACR measurement, concurrent with a drop in UACR category between A1 (<3 mg/mmol), A2 (3–29 mg/mmol)) or A3 (≥30 mg/mmol). eGFR and UACR trajectories following DD2 enrolment without any CKD prior to enrolment were used to model kidney function following type 2 diabetes diagnosis. To exclude eGFR and UACR values that may represent acute manifestations of kidney dysfunction (such as acute infections or dehydration) rather than CKD, we excluded all measurements obtained from emergency departments or during inpatient hospital admissions. ESM Table 3 provides further details regarding outcome definitions.

### Statistical analyses

Descriptive data for continuous variables were summarised as medians and IQRs, while categorical variables were described using counts and percentages, according to the birthweight categories, i.e. clinical cut-offs and percentile groups. Participants were followed from DD2 enrolment until the first occurrence of a CKD outcome, death, emigration or the end of follow-up (12 March 2024), whichever occurred first. The median follow-up duration was calculated from the date of enrolment to the earliest of these events. To address missing data, multivariate imputations by chained equations (MICE) was performed using the mice package (version 3.14.0) in R [22]. The proportions of missing values ranged from 0% to 56% (median 12% among covariates with missing values), with the highest proportion being for blood pressure. The pattern of missingness was consistent across birthweight categories, sex and ages at enrolment. Additional specifications regarding the MICE model are provided in ESM Methods.

All analyses were performed using R software version 4.1.2 (R Project for Statistical Computing). This study adheres to the Strengthening the Reporting of Observational Studies in Epidemiology (STROBE) guidelines.

#### Hazard ratio

We calculated cause-specific HRs as a measure of the incidence rate ratio using Cox proportional hazard models. Models were adjusted for the following potential confounders: sex, age at enrolment, calendar year of birth, family history of type 2 diabetes, and born-at-term status. As birthweight is an exposure that is defined at the time of birth, no further adjustments were made in our main model (to avoid over-adjustment). We considered later-life socio-behavioural, metabolic and lifestyle factors as potential intermediate variables between birthweight, type 2 diabetes and later CKD. Additional exploratory analyses included adjustments for behavioural lifestyle factors (physical activity, smoking status and alcohol consumption), socioeconomic markers (marital status and rural/urban residence), BMI, number of antihypertensive medications (as a proxy for hypertension burden), number of glucose-lowering medications (as a proxy for the dysmetabolic state in type 2 diabetes), and polygenic risk scores for type 2 diabetes, birthweight and CKD. The proportional hazards assumption was verified using Schoenfeld or Martingale residual plots, with age at enrolment categorised for model stability. Finally, in a Cox regression analysis, we modelled birthweight as a continuous exposure using linear and restricted cubic spline functions [23], with a reference median birthweight of 3400 g (HR=1).

#### Absolute risk

Using the Aalen–Johansen estimator, we calculated standardised 10-year absolute risk estimates for incident CKD and CKD progression, with death considered as the competing risk. To account for potential confounders, we used the parametric G-formula, analogous to direct standardisation [24, 25]. This involved fitting two cause-specific Cox regression models: one for the hazard rates of the outcome and another for the competing risks. Using the aforementioned Cox models and the G-formula [26], we calculated the average absolute risk for each birthweight category, standardised to the confounder distribution of the entire cohort. Standardised risk differences (RDs) were estimated by contrasting the risks in the low and high birthweight groups with those in the reference group. These standardised absolute risks and RDs represent weighted averages of the conditional averages within each confounder stratum. Non-parametric bootstrapping with 10,000 samples was used to obtain 95% CIs [24]. All models were standardised by sex, age at enrolment, calendar year of birth, family history of type 2 diabetes, and born-at-term status.

#### Mixed-effects models

Mixed-effects models were used to understand separate trajectories of eGFR and UACR following enrolment, based on birthweight categories (clinical cut-offs, percentiles and as a continuous variable). Repeated measurements of eGFR/UACR since enrolment served as dependent variables. Fixed effects included time from enrolment to each eGFR/UACR measurement, birthweight categories and potential confounders (sex, age at enrolment, calendar year at birth, family history of type 2 diabetes, and born-at-term status). To account for association between a low eGFR/high UACR measurement and the associated increased number of repeated eGFR/UACR measurements (and thus shorter time between measurements), we included a random effect for time from enrolment to each eGFR/UACR measurement for individual participants. UACR values were log-transformed, with the results presented as a percentage change in UACR. Linear and restricted cubic spline functions were applied to time from enrolment to eGFR/UACR measurement.

#### Sensitivity analysis

We calculated sub-distributional adjusted hazard ratios (aHRs) using the Fine–Gray model, with death considered as the competing risk. To mitigate potential misclassification of prevalent CKD cases, we performed a sensitivity analysis excluding individuals with CKD who were diagnosed within 6 months of DD2 enrolment (*n*=48). Stratified analyses were conducted by sex, restriction to individuals born at term and to individuals without a prior history of CVD. Finally, we investigated the risk of incident CKD based on eGFR or UACR separately to explore differences between these two measures.

### Ethics

This study was approved by the Danish Data Protection Agency (record number 2008-58-0035) and by the Regional Committees on Health Research Ethics for Southern Denmark (record number S-20100082). All cohort participants gave written informed consent.

## Results

The DD2 cohort enrolled 11,375 participants during the 2010–2024 period. Because of the incomplete coverage of the National Laboratory Database until 2015, we excluded 2593 participants (23%) who lacked laboratory measurements within 2 years prior to enrolment. Of the 8782 participants with available laboratory data, 337 (3.8%) were excluded because the eGFR measurements needed to establish baseline kidney function were missing. Of the remaining 8445 participants, 2297 (27%) had incomplete birth records, with missing birthweight data for the reasons explained previously [7]. This left 6148 participants with complete midwife records. After excluding 74 people with positive GAD antibodies (>30 kU/l) (to avoid potential misclassification of autoimmune diabetes as type 2 diabetes) and 92 with non-singleton births, our final analytic cohort included 5982 participants (ESM Fig. 1). Among them, 427 (7%) had a birthweight <2500 g, 4999 (84%) had a birthweight of 2500–4000 g, and 556 (9%) had a birthweight >4000 g. For birthweight categories based on quartiles, 1554 (26%) had a birthweight <3000 g, 3049 (51%) had a birthweight of 3000–3700 g, and 1379 (23%) had a birthweight >3700 g. Table 1 and ESM Table 4 show the baseline characteristics according to birthweight category. Participants with a birthweight <2500 g were 5.8 years younger than the median age at enrolment, were more likely to be of female sex and less likely to be married or in a registered partnership. As previously reported, participants with LBW were associated with a lower BMI, having fewer relatives with type 2 diabetes, and with greater use of glucose-lowering medication and antihypertensive medication than particiapants with a birthweight of 2500–4000 g [7]. Further detailed information on medication usage is provided in ESM Tables 5 and 6.

**Table 1:** Baseline characteristics at enrolment according to birthweight of people recently diagnosed with type 2 diabetes

Variable	<2500 g (*n*=427)	2500–4000 g (*n*=4999)	>4000 g (*n*=556)	Total (*n*=5982)
Sex				
Female	203 (47.5)	2040 (40.8)	162 (29.1)	2405 (40.2)
Age at DD2 enrolment (years)				
Median (IQR)	54.5 (46.9, 63.0)	60.5 (51.1, 67.9)	62.5 (52.0, 69.4)	60.3 (50.7, 67.8)
<45	79 (18.5)	589 (11.8)	69 (12.4)	737 (12.3)
45, 55	141 (33.0)	1157 (23.1)	114 (20.5)	1412 (23.6)
55, 65	118 (27.6)	1506 (30.1)	134 (24.1)	1758 (29.4)
65, 75	67 (15.7)	1369 (27.4)	183 (32.9)	1619 (27.1)
>75	22 (5.2)	378 (7.6)	56 (10.1)	456 (7.6)
Family history of type 2 diabetes^a^				
0	206 (48.2)	2445 (48.9)	249 (44.8)	2900 (48.5)
1	120 (28.1)	1545 (30.9)	178 (32.0)	1843 (30.8)
2	66 (15.5)	745 (14.9)	94 (16.9)	905 (15.1)
3+	35 (8.2)	264 (5.3)	35 (6.3)	334 (5.6)
Marital/civil status				
Married/partnership	214 (52.5)	2889 (59.6)	331 (61.0)	3434 (59.2)
Divorced/separated	83 (20.3)	826 (17.0)	85 (15.7)	994 (17.1)
Widow/widower	19 (4.7)	369 (7.6)	43 (7.9)	431 (7.4)
Not married/no registered partnership	92 (22.5)	762 (15.7)	84 (15.5)	938 (16.2)
Missing	19	153	13	185
Rural/urban residence				
Capital municipalities	79 (18.5)	969 (19.4)	94 (16.9)	1142 (19.1)
Large city municipalities	81 (19.0)	1126 (22.5)	108 (19.4)	1315 (22.0)
Provincial municipalities	120 (28.1)	1223 (24.5)	141 (25.4)	1484 (24.8)
Surrounding area municipalities	72 (16.9)	878 (17.6)	112 (20.1)	1062 (17.8)
Rural area municipalities	75 (17.6)	803 (16.1)	101 (18.2)	979 (16.4)
Born-at-term status				
Preterm	312 (73.1)	534 (10.7)	34 (6.1)	880 (14.7)
BMI (kg/m^2^)				
Median (IQR)	31.0 (27.9, 34.9)	31.2 (27.7, 35.8)	31.7 (28.1, 36.4)	31.3 (27.8, 35.8)
<25	33 (12.5)	347 (11.0)	28 (8.1)	408 (10.9)
25, 30	80 (30.4)	940 (29.9)	112 (32.6)	1132 (30.2)
30, 35	86 (32.7)	953 (30.3)	94 (27.3)	1133 (30.2)
35, 40	35 (13.3)	516 (16.4)	64 (18.6)	615 (16.4)
>40	29 (11.0)	385 (12.3)	46 (13.4)	460 (12.3)
Missing	164	1858	212	2234
Waist circumference (cm)				
Median (IQR)	107 (100, 116)	108 (102, 117)	110 (103, 120)	108 (102, 117)
Missing	12	99	16	127
Alcohol consumption^b^				
>14/21 units per week	15 (3.5)	297 (5.9)	29 (5.2)	341 (5.7)
Smoking status				
Never	94 (55.0)	1059 (46.9)	103 (42.0)	1256 (46.9)
Former	47 (27.5)	788 (34.9)	80 (32.7)	915 (34.2)
Current	30 (17.5)	413 (18.3)	62 (25.3)	505 (18.9)
Missing	256	2739	311	3306
Physical activity (days per week)				
0	56 (13.1)	667 (13.3)	70 (12.6)	793 (13.3)
1 or 2	100 (23.4)	1068 (21.4)	104 (18.7)	1272 (21.3)
3 or 4	103 (24.1)	1160 (23.2)	135 (24.3)	1398 (23.4)
5 or 6	75 (17.6)	854 (17.1)	87 (15.6)	1016 (17.0)
7	93 (21.8)	1250 (25.0)	160 (28.8)	1503 (25.1)
Systolic BP (mmHg)				
Median (IQR)	131 (124, 142)	132 (125, 141)	130 (122, 142)	131 (125, 141)
Missing	259	2773	310	3342
Diastolic BP (mmHg)				
Median (IQR)	82 (76, 89)	80 (75, 88)	80 (75, 88)	80 (75, 88)
Missing	259	2773	310	3342
Total cholesterol (mmol/l)				
Median (IQR)	4.3 (3.7, 5.2)	4.3 (3.7, 5.1)	4.3 (3.6, 5.0)	4.3 (3.7, 5.1)
Missing	51	645	57	753
Triglycerides (mmol/l)				
Median (IQR)	1.9 (1.3, 2.7)	1.8 (1.2, 2.6)	1.7 (1.2, 2.4)	1.8 (1.2, 2.6)
Missing	38	547	51	636
HDL-cholesterol (mmol/l)				
Median (IQR)	1.1 (0.9, 1.3)	1.1 (1.0, 1.4)	1.1 (0.9, 1.3)	1.1 (1.0, 1.4)
Missing	51	657	57	765
LDL-cholesterol (mmol/l)				
Median (IQR)	2.2 (1.7, 2.9)	2.2 (1.7, 2.9)	2.2 (1.7, 2.9)	2.2 (1.7, 2.9)
Missing	43	584	56	683
Blood glucose (mmol/l)				
Median (IQR)	7.3 (6.4, 8.4)	7.3 (6.4, 8.5)	7.2 (6.2, 8.5)	7.3 (6.4, 8.5)
Missing	63	531	61	655
HbA_1c_ (mmol/mol)				
Median (IQR)	49 (44, 56)	48 (44, 55)	49 (44, 56)	48 (44, 55)
HbA_1c_ (%)				
Median (IQR)	6.6 (6.2, 7.3)	6.5 (6.2, 7.2)	6.6 (6.2, 7.3)	6.5 (6.2, 7.2)
Missing	2	66	5	73
C-peptide (pmol/l)				
Median (IQR)	1240 (900, 1690)	1220 (910, 1660)	1210 (870, 1650)	1220 (910, 1660)
Missing	63	526	61	650
HOMA-2B				
Median (IQR)	100.6 (66.7, 130.8)	94.1 (70.5, 124.1)	96.4 (69.9, 128.2)	94.6 (70.2, 125.1)
Missing	71	634	70	775
HOMA2-IR				
Median (IQR)	32.1 (24.0, 44.7)	32.6 (24.3, 44.6)	33.0 (24.1, 45.8)	32.6 (24.3, 44.8)
Missing	71	634	70	775
hsCRP (mg/l)				
Median (IQR)	2.2 (0.9, 4.7)	2.1 (0.9, 4.6)	2.0 (0.8, 4.4)	2.1 (0.9, 4.6)
Missing	174	1770	192	2136
PRS for T2D (scaled)				
Median (IQR)	−0.2 (−0.7, 0.6)	0.0 (−0.7, 0.7)	−0.1 (−0.7, 0.7)	−0.0 (−0.7, 0.7)
Missing	55	497	66	618
PRS for birthweight				
Median (IQR)	−0.2 (−0.9, 0.5)	−0.0 (−0.7, 0.6)	0.4 (−0.2, 1.0)	0.0 (−0.7, 0.7)
Missing	55	497	66	618
PRS for CKD				
Median (IQR)	−0.1 (−0.7, 0.7)	0.0 (−0.7, 0.7)	−0.1 (−0.7, 0.6)	−0.0 (−0.7, 0.7)
Missing	55	497	66	618

Values are given as *n* (%) unless otherwise indicated

^a^Number of relatives with type 2 diabetes

^b^Using limits of 14 units per week for women and 21 units per week for men

hsCRP, high-sensitivity C-reactive protein; PRS, polygenic risk score; T2D, type 2 diabetes

### Incident CKD

A total of 1501 incident CKD outcomes (incidence rate 42.4 per 1000 person-years) and 768 deaths (incidence rate 16.9 per 1000 person-years) occurred during the median follow-up period of 8.3 years (IQR 4.8–10.3 years) among the 5393 people with type 2 diabetes and without a history of CKD at enrolment (ESM Tables 7 and 8).

**Fig. 1:**
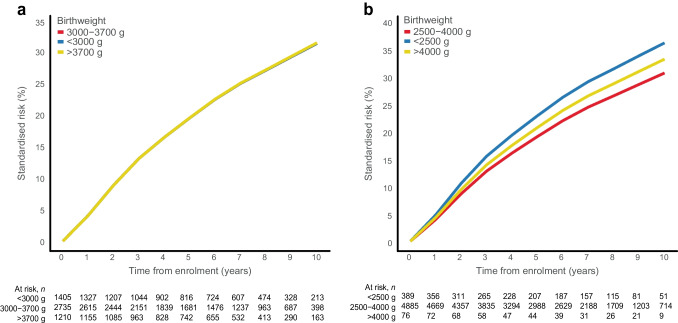
Absolute risk of incident CKD by birthweight categories, showing risk curves for incident CKD according to birthweight. The absolute risk curves were derived from a model standardised to the distribution of sex, age at DD2 enrolment, calendar year of birth, family history of type 2 diabetes, and born-at-term status. (**a**) Percentile-based birthweight categories. (**b**) Clinical birthweight categories

**Fig. 2:**
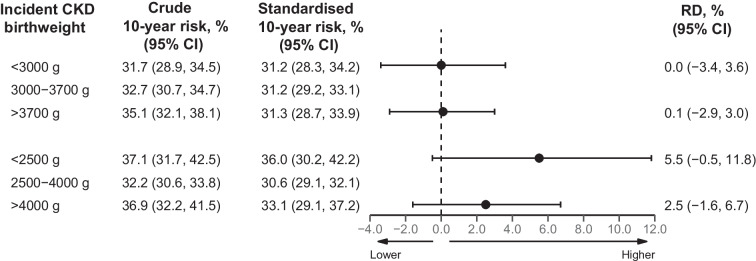
Ten-year standardised risk and RD for incident CKD according to birthweight categories. Reference birthweight (3000–3700 g for percentile-based birthweight category and 2500–4000 g for clinical birthweight category) were compared with the lower birthweight categories (<3000 g and <2500 g, respectively) and the higher birthweight categories (>3700 g and >4000 g, respectively). The Forest plot was derived from a model standardised to the distribution of sex, age at DD2 enrolment, calendar year of birth, family history of type 2 diabetes, and born-at-term status

**Fig. 3:**
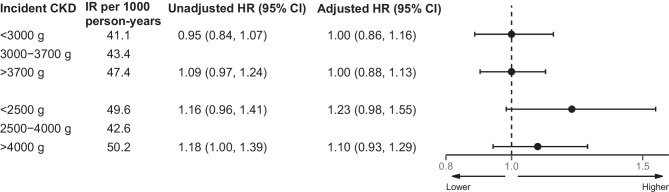
Cause-specific Cox regression of incident CKD and incidence rates per 1000 person-years according to birthweight categories, showing HRs for incident CKD according to birthweight. The reference birthweight groups (3000–3700 g for percentile-based birthweight category and 2500–4000 g for clinical birthweight category) were compared with the lower birthweight categories (<3000 g and <2500 g, respectively) and the higher birthweight categories (>3700 g and >4000 g, respectively), adjusted for sex, age at DD2 enrolment, calendar year of birth, family history of type 2 diabetes, and born-at-term status. IR, incidence rate

The 10-year standardised CKD risk was highest in those with a birthweight <2500 g (36.0%, compared to 30.6% for those with a birthweight of 2500–4000 g), yielding a 10-year standardised RD of 5.5% (95% CI −0.5%, 11.8%), corresponding to an aHR of 1.23 (95% CI 0.98, 1.55) (ESM Tables 9 and 10, Fig. 1b, Fig. 2 and Fig. 3). For birthweights >4000 g vs 2500–4000 g, the 10-year standardised CKD risk was slightly higher (33.1% vs 30.6%, respectively), yielding an RD of 2.5% (95% CI −1.6%, 6.7%) and an aHR of 1.10 (95% CI 0.93, 1.29) (ESM Tables 9 and 10, Fig. 1b, Fig. 2, and Fig. 3). Notably, the HR in those with birthweight <2500 g compared with 2500–4000 g increased from 1.16 (95% CI 0.96, 1.41) to 1.36 (95% CI 1.12, 1.65) after adjusting for the fact that female sex and younger age were more frequent in those with a birthweight <2500 g (ESM Table 10). After further adjusting for preterm birth, the aHR for a birthweight <2500 g decreased to 1.23 (95% CI 0.98, 1.55) (ESM Table 10). Similar risk associations were observed after adjusting for physical activity, smoking status, alcohol consumption, marital status, rural/urban residence, BMI, number of antihypertensive and glucose-lowering medications, and polygenic risk scores for type 2 diabetes, birthweight and CKD (ESM Table 10).

For percentile-defined birthweight groups, the 10-year standardised CKD risk was similar in people with a birthweight <3000 g (31.2%) and 3000–3700 g (31.2%), yielding an RD of 0.0% (95% CI −3.4%, 3.6%) and an aHR of 1.00 (95% CI 0.86, 1.16) (ESM Tables 9 and 10, Fig. 1a, Fig. 2, and Fig. 3). Likewise, birthweights >3700 g and 3000–3700 g showed similar 10-year standardised CKD risks (31.3% vs 31.2%, respectively), with an aHR of 1.00 (95% CI 0.88, 1.13) (ESM Tables 9 and 10, Fig. 1a, Fig. 2, and Fig. 3). Further adjustments did not materially change these associations (ESM Table 10).

### Progression of incident CKD

**Fig. 4:**
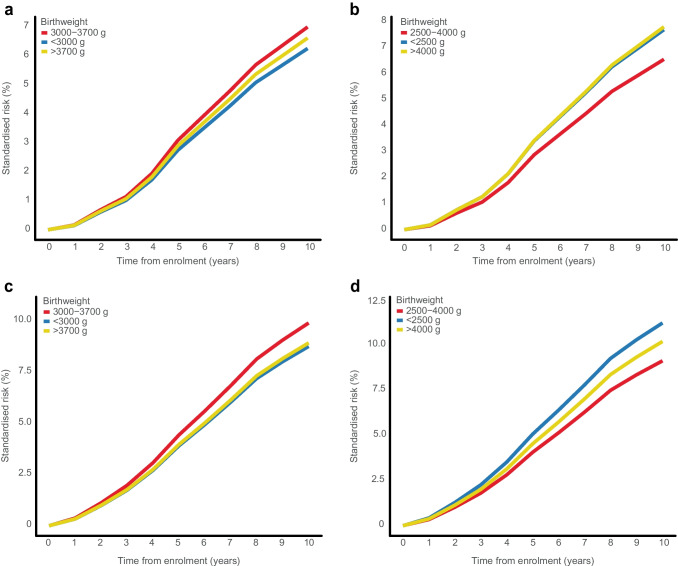
Standardised risk curves for CKD progression (eGFR-based and eGFR + UACR combined), according to birthweight groups. The absolute risk curves were derived from a model standardised to the distribution of sex, age at DD2 enrolment, calendar year of birth, family history of type 2 diabetes, and born-at-term status. (**a**) Results for percentile-based birthweight categories with CKD progression based only on eGFR. (**b**) Results for clinical birthweight categories with CKD progression based only on eGFR. (**c**) Results for percentile-based birthweight categories with CKD progression based on eGFR and UACR combined. (**d**) Results for clinical birthweight categories with CKD progression based on eGFR and UACR combined

During follow-up, 543 people (incidence rate 11.0 per 1000 person-years) fulfilled the criteria for eGFR-defined CKD progression among the 5393 people with type 2 diabetes and without a history of CKD at enrolment (ESM Tables 7 and 8). The 10-year standardised CKD progression risk was slightly higher in people with a birthweight <2500 g (7.7%) compared to those with a birthweight of 2500–4000 g (6.5%), corresponding to an RD of 1.1% (95% CI −2.1%, 5.0%) and an aHR of 1.19 (95% CI 0.71, 2.01) (ESM Tables 11 and 12, Fig. 4). Similarly, for birthweights >4000 g vs 2500–4000 g, the 10-year standardised CKD risk was also slightly higher (7.8% vs 6.5%), corresponding to an RD of 1.2 (95% CI −1.1%, 3.8%) and an aHR of 1.22 (95% CI 0.86, 1.72) (ESM Tables 11 and 12, Fig. 4). Further adjustments did not materially change these associations (ESM Table 12).

For percentile-defined birthweight groups, the 10-year standardised CKD progression risk was slightly lower in people with a birthweight <3000 g (6.2%) compared to those with a birthweight of 3000–3700 g (7.0%), yielding an RD of −0.7% (95% CI −2.6%, 1.2%) and an aHR of 0.89 (95% CI 0.64, 1.24) (ESM Tables 11 and 12, Fig. 4). For birthweights >3700 g vs 3000–3700 g, the 10-year standardised CKD risk was also slightly lower (6.6% vs 7.0%, respectively), with an aHR of 0.93 (95% CI 0.72, 1.21) (ESM Tables 11 and 12, Fig. 4). Further adjustments did not materially change the associations (ESM Table 12). Including UACR in the CKD progression outcome definition, increased the 10-year standardised CKD progression risk to 11.2% for birthweights <2500 g vs 9.1% for birthweights 2500–4000 g, yielding an RD of 2.1% (95% CI −1.9%, 6.9%) and an aHR of 1.58 (95% CI 0.82, 3.05) (ESM Tables 11 and 12, Fig. 4).

### Continuous birthweight analyses

For incident CKD, all models showed similar patterns of increasing aHR with lower birthweight, using the median birthweight of 3400 g as the reference (ESM Fig. 2). Compared with linear models, spline models showed a larger increase in aHR with lower birthweight (ESM Fig. 2). For CKD progression based on eGFR, the linear model showed a slight decrease in aHR with decreasing birthweight, while spline models revealed a pattern similar to that for incident CKD (ESM Fig. 2). When combining eGFR with UACR for CKD progression, the aHR also increased as birthweight decreased (ESM Fig. 2).

### Mixed-effects models

#### eGFR

**Fig. 5:**
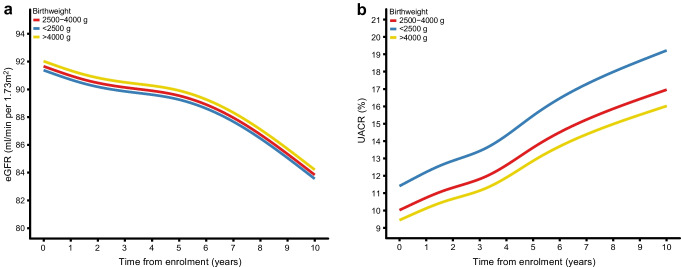
Mixed-effects models of eGFR and UACR trajectories according to clinical birthweight categories. Repeated measures of eGFR/UACR since enrolment were used as the dependent variable. Fixed effects were time from enrolment until eGFR/UACR measurement, birthweight categories, and confounders (sex, age at enrolment, calendar year of birth, family history of type 2 diabetes, and born-at-term status). Random effects were time from enrolment until eGFR/UACR measurement for individual participants. Restricted cubic splines were used for time from enrolment to eGFR/UACR measurement. UACR was log-transformed, and estimates are represented as the % change in UACR. (**a**) Results for eGFR trajectories. (**b**) Results for UACR trajectories

Among the 5376 people with at least one eGFR measurement after enrolment and no prior CKD, the median number of eGFR measurements per person was 19 (IQR 11–30), with a median of three measurements (IQR 2.1–4.1) per year of follow-up. The number of measurements per year was consistent across all birthweight categories (ESM Table 13). No models showed any large differences in eGFR trajectories for either continuous or categorical birthweight exposures (ESM Table 13, Fig. 5).

#### UACR

For UACR, 5200 participants had at least one measurement after enrolment and no prior CKD, with a median of six measurements per person (IQR 4–9), and 1.2 (IQR 1.0–1.6) per year of follow-up. The measurement frequency was similar across birthweight categories (ESM Table 8). In adjusted mixed-effects spline models with four knots, each 1 kg decrease in birthweight was associated with a 6.6% (95% CI 1.9%, 11.1%) higher UACR at baseline, with similar slopes (ESM Table 13). A birthweight <2500 g, compared with 2500–4000 g, was associated with a 12.6% (95% CI 1.2%, 25.3%) higher UACR at baseline. However, the slope was comparable to that for people with a birthweight of 2500–4000 g (ESM Table 13, Fig. 5). For birthweights >4000 g vs 2500–4000 g, the UACR was 5.2% (95% CI −16.4, 22.8) lower, although the estimates were statistically imprecise with wide 95% CI (ESM Table 13, Fig. 5). Birthweights <3000 g and >3700 g did not show a large difference in UACR compared with 3000–3700 g (ESM Table 13).

### Sensitivity analysis

The Fine–Gray model showed associations comparable to those from cause-specific Cox models for incident CKD (ESM Table 14). Excluding CKD cases diagnosed within 6 months before enrolment did not attenuate the associations (ESM Table 15). In sex-stratified analyses, the risk of incident CKD for birthweights <2500 g vs 2500–4000 g was similar between sexes. However, for birthweights >4000 g vs 2500–4000 g, male participants had a 10-year standardised RD of 4.1% (95% CI −0.9%, 9.1%), while female participants had an RD of −0.5% (95% CI −8.1%, 7.5%). This corresponded to an aHR of 1.17 (95% CI 0.96, 1.42) for male participants and an aHR of 0.96 (95% CI 0.69, 1.33) for female participants (ESM Tables 16 and 17, ESM Fig. 3). Use of cause-specific Cox models with splines for continuous birthweight exposure mirrored the findings for the clinical birthweight category (ESM Fig. 2).

When assessing incident CKD risk using only eGFR or UACR measurements separately, the associations were similar to the main analysis for low birthweights, i.e. <2500 g vs 2500–4500 g. The 10-year standardised RD for incident CKD based on UACR was approximately double that for eGFR (RD 4.1% [95% CI −1.0%, 9.4%] and 2.4% [95% CI −2.2%, 7.4%], respectively) (ESM Table 18). For birthweights >4000 g vs 2500–4000 g, the 10-year standardised RD for eGFR was 4.0% (95% CI 0.6%, 7.4%), with an aHR of 1.28 (95% CI 1.04, 1.57) (ESM Tables 18 and 19). Restricting the analysis to those born at term and without pre-existing CVD yielded similar estimates to the non-stratified analyses (ESM Tables 20 and 21).

## Discussion

In this prospective study of 5982 people with recently diagnosed type 2 diabetes, a birthweight <2500 g was associated with elevated risk of incident CKD, with parameter values ranging from no effect to a considerable increase in risk. Continuous spline models revealed a pattern of increasing aHR with decreasing birthweight. Trajectory modelling revealed an association between LBW and higher UACR, but no association with eGFR.

To our knowledge, this is the first prospective study investigating the relationship between birthweight and incident CKD with trajectory modelling of kidney function among people with new-onset type 2 diabetes. This is clinically important as those with type 2 diabetes already face an elevated baseline risk of CKD. Those with the lowest birthweights are characterised by earlier diabetes onset, more hypertension and greater overall comorbidity at diagnosis, together with an increased risk of CVD following diabetes onset [7, 8]. While elevated CKD risk among LBW people with type 2 diabetes was associated with parameter values ranging from no effect to a considerable increase in risk, these findings add to emerging evidence that LBW individuals with type 2 diabetes have excess risk of type 2 diabetes complications and comorbidities.

Consistent with a smaller study in Pima Indians [11], we observed elevated UACR in LBW individuals with type 2 diabetes. While a U-shaped association between birthweight and CKD risk was reported among Pima Indians, we found this pattern only among male participants. Further research is needed to understand the role of sex in the relationship between high birthweight and CKD risk.

Adjusting for preterm birth reduced the CKD risk associated with LBW by 13%. As preterm birth, being small for gestational age and LBW are all terms that may be used to describe ‘small vulnerable newborns’ [27], with shared risk factors, causes and health consequences, adjusting for one (preterm birth) while studying the impact of another (LBW) may lead to over-adjustment. Sensitivity analyses restricted to those born at term produced similar estimates to non-stratified analyses.

Putative mechanisms underlying the observed associations between an adverse intrauterine environment and CKD include lower nephron numbers, smaller glomerular volume and accelerated kidney disease progression [2, 3]. The kidneys have a continuous age-dependent loss of nephrons [28], which is mitigated by compensatory hyperfiltration within the remaining nephrons [29]. However, this compensatory hyperfiltration may result in intraglomerular hypertension, increased shear stress and expansion of the glomerular basement membrane, eventually causing podocyte detachment and glomerular sclerosis [30]. These adaptations may make LBW individuals more susceptible to CKD, especially when combined with secondary insults such as diabetes, obesity or hypertension, suggesting a possible ‘two-hit model’ [31–33].

We found an almost doubled risk of incident CKD when using UACR compared with eGFR. The explanation for this is uncertain, but may reflect increased intraglomerular pressure and albumin filtration into the urine due to a lower number of nephrons and hence fewer functional glomerular units in individuals with LBW [2, 3]. UACR is generally recognised as a more sensitive marker of early kidney damage compared with eGFR [34]. This may be explained by compensatory glomerular hypertrophy sustaining the GFR at a normal or even elevated level during the earliest stages of CKD development [3]. Notably, previous animal studies have documented that prenatal undernourishment is associated with smaller kidneys and reduced nephron numbers [35, 36]. Autopsy studies have similarly reported reduced nephron numbers in humans with LBW as well as preterm birth [37, 38]. Collectively, our study suggests a scenario in which LBW is causally associated with a more immature structure and/or functionality of the adult kidney, reflected by fewer functional glomerular units. These changes appears to be present at the onset of type 2 diabetes, and, to some extent, is independent of elevated plasma glucose levels, which are otherwise considered to be a primary driver of diabetic nephropathy [39]. This discrepancy may also be related to prenatal environmental factors affecting muscle development [40, 41]. As most circulating creatinine derives from muscle, low muscle mass may distort CKD risk assessments, leading to overestimation of both eGFR [42] and UACR [43]. Such overestimation of eGFR may lead to underestimation of CKD risk, while overestimation of UACR may conversely lead to overestimation of CKD risk. While BMI adjustments did not alter our findings, BMI may not fully account for muscle mass differences and further studies are needed to address this issue.

Trajectory modelling of kidney function indicated that, although LBW was associated with elevated UACR at baseline, the difference did not increase over time. This suggests that the effect of LBW on kidney function, as reflected by UACR, is particularly evident during the early stages of type 2 diabetes but does not increase during progression of the disease. This may potentially be attributed to lower nephron numbers and a constitutively reduced glomerular volume in LBW individuals that already exists at the time of type 2 diabetes onset [2, 3, 31–33]. This is clinically relevant and raises questions about the timing of interventions, to enable elevated UACR to be treated at the earliest possible time point. Further research is needed to understand these mechanisms and to identify effective interventions for the high-risk group of people with elevated UACR.

The strengths of this nationwide study include its well-characterised cohort of participants who were recently diagnosed with type 2 diabetes, recruited from both primary and secondary healthcare. Robust data linkage to high-quality health registries provided access to laboratory-measured eGFR and UACR as indicators of kidney function. Recall bias was minimised through use of objectively ascertained birthweight from original midwife records dating back nearly a century. Further adjustments for genetic predispositions for type 2 diabetes, birthweight and CKD did not attenuate the findings.

Study limitations include potential survival and selection biases before entering the DD2 cohort, probably reducing participation among those at high risk of CKD or with LBW, leading to underestimation of CKD rates and biasing results toward the null. LBW is also a known risk factor for multiple disorders – such as cardiovascular, lung, mental and neurological diseases – that may precede type 2 diabetes and CKD [40, 44–47]. We lacked data on several intermediate factors from birth through adulthood, including early socio-behavioural factors, education, occupation and income. These factors may act as mediators of LBW, type 2 diabetes and CKD, or reflect unmeasured confounders among parents, leading to both LBW and later type 2 diabetes and CKD in offspring. While confounding by indication may affect UACR measurements due to selective testing of high-risk individuals, this is unlikely given that Danish guidelines recommend annual UACR screening for type 2 diabetes [48], and we took this into account as a random effect in our trajectory modelling. Data were missing for some covariates. Our main model included only complete covariate data; missing data were addressed using multiple imputation in exploratory models. The study population was probably homogeneous in terms of race/ethnicity due to the demographic at the time when participants were born in Denmark, limiting generalisability and necessitating further research across diverse populations. Finally, LBW may not be the direct causal factor for increased CKD risk in people with type 2 diabetes, but rather serve as a marker for various adverse fetal exposures, including maternal nutrition, health status, smoking, medication, stress, socioeconomic and/or psychological variables. These exposures can affect long-term organ structure and function [49], making birthweight a proxy for multiple early-life determinants of later CKD risk in people with type 2 diabetes.

### Conclusion

Despite limited precision, our study indicates that LBW is associated with disproportionately increased risk of incident CKD among people with recently diagnosed type 2 diabetes. The finding adds further support to the emerging evidence of LBW as an early-life marker for a more severe disease course in type 2 diabetes, which besides CKD includes earlier onset of diabetes, a larger burden of hypertension, and increased morbidity and mortality risk from cardiovascular disease.

Further studies are needed to understand the extent to which birthweight information should be implemented to guide clinical care and reduce cardiovascular as well as renal comorbidities in type 2 diabetes.

## Supplementary Information

Below is the link to the electronic supplementary material.

ESM (PDF 1289 KB)

## Data Availability

Danish data protection legislation does not allow sharing of the individual-level personal data used for this study. However, a data dictionary for variables used in the study and analysis code is in preparation and will be shared and made publicly available on the DD2 website, www.dd2.dk when available. Requests to access the Danish health registries used in this study may be sent from researchers at authorised research institutions to the Danish Health Data Authority by email (forskerservice@sundhedsdata.dk). Requests to use the primary DD2 data may be made by email using the link at https://dd2.dk/forskning/ansoeg-om-data.

## Abbreviations

aHR: Adjusted hazard ratio
CKD: Chronic kidney disease
DD2: Danish Centre for Strategic Research in Type 2 Diabetes
LBW: Low birthweight
RD: Risk difference
UACR: Urine albumin/creatinine ratio

## Authors’ relationships and activities

CB owns stock in Novo Nordisk. MHO has received payments or honoraria for lectures, presentations or educational events from AstraZeneca and Boehringer Ingelheim, has an unpaid position as chair of the Danish Hypertension Society, and is a nucleus member of the Working Group for Prevention and Rehabilitation, Danish Society of Cardiology. None of these studies have any relation to the present study. The authors declare that there are no other relationships or activities that might bias, or be perceived to bias, their work.
