# Supplementary material for: Birthweight and risk of chronic kidney disease after a type 2 diabetes diagnosis in the DD2 cohort
Source: Diabetologia. 2025 Feb 1;68(4):778–91. doi: 10.1007/s00125-024-06357-4 (PMC11950141; doi:10.1007/s00125-024-06357-4)
Supplement: Supplementary file 1 — ESM (PDF 1289 KB) [file 125_2024_6357_MOESM1_ESM.pdf]

Electronic supplementary material:

Table of contents

Electronic supplementary material: 1

ESM Methods: 2

ESM Method: Multivariate imputations by chained equations (MICE) model specification 2

ESM Table 1: data sources 7

ESM Table 2: Variable definitions 8

ESM Table 3: Outcome definitions 14

ESM Table 4: Baseline characteristics at enrolment according to conventional birthweight categories 15

ESM Table 5: Medication according to birthweight categories 18

ESM Table 6: Medication according to birthweight categories 19

ESM Table 7: Incident and prior CKD according to birthweight categories 20

ESM Table 8: Incident and prior CKD according to conventional birthweight categories 20

ESM Table 9: 10-year standardized risk and standardized risk difference estimates for incident CKD according to birthweight categories 20

ESM Table 10: Stepwise adjustments in cause-specific Cox proportional hazard regression of incident CKD, according to birthweight categories. 21

ESM Table 11: 10-year standardized risk and standardized risk difference estimates for CKD progression according to birthweight categories. 22

ESM Table 12: Stepwise adjustments in cause-specific Cox proportional hazard regression of CKD progression, according to birthweight categories. 22

ESM Table 13: eGFR and UACR repeated measurements and mixed effects models according to birthweight categories. 24

ESM Table 14: Sub distributional hazard ratios from Fine-Gray models for incident CKD, according to birthweight. 24

ESM Table 15: Cause-specific Cox proportional hazard regression of incident CKD by birthweight, excluding CKD cases diagnosed within six months before DD2 enrolment. 25

ESM Table 16: Sex stratified 10-year standardized risk and standardized risk difference estimates for incident CKD according to birthweight categories 25

ESM Table 17: Sex-stratified stepwise adjustments in cause-specific Cox proportional hazard regression of incident CKD, according to birthweight categories. 25

ESM Table 18: 10-year standardized risk and standardized risk difference estimates for incident CKD based on only eGFR or only UACR, according to birthweight categories. 26

ESM Table 19: Cause-specific Cox proportional hazard regression of incident CKD based on only eGFR or only UACR, according to birthweight categories. 27

ESM Table 20: Cause-specific Cox proportional hazard regression of incident CKD without pre-existing cardiovascular disease, according to birthweight categories. 27

ESM Table 21: Cause-specific Cox proportional hazard regression of incident CKD restricting to individuals born-at-term, according to birthweight categories. 27

ESM Fig. 1: Flowchart of study population. 29

ESM Fig. 2: Birthweight as a continuous exposure using restricted cubic spline regression with Cox proportional hazard regression. 30

ESM Fig. 3: Sex-stratified standardized risk for incident CKD according to birthweight groups. 35

ESM Methods:

ESM Method: Multivariate imputations by chained equations (MICE) model specification

We employed multivariate imputations by chained equations using the MICE package from R[1]. The percentage of missing values across the dataset ranged from 0 to 56%, with an average of 7% missing values across all variables. Included below are plots showing the percentage of missing data per variable according to birthweight, age at enrolment, and sex. The plots show that the distributions of missing data according to birthweight categories, age at enrolment, and sex were similar, providing evidence against potential attrition bias.

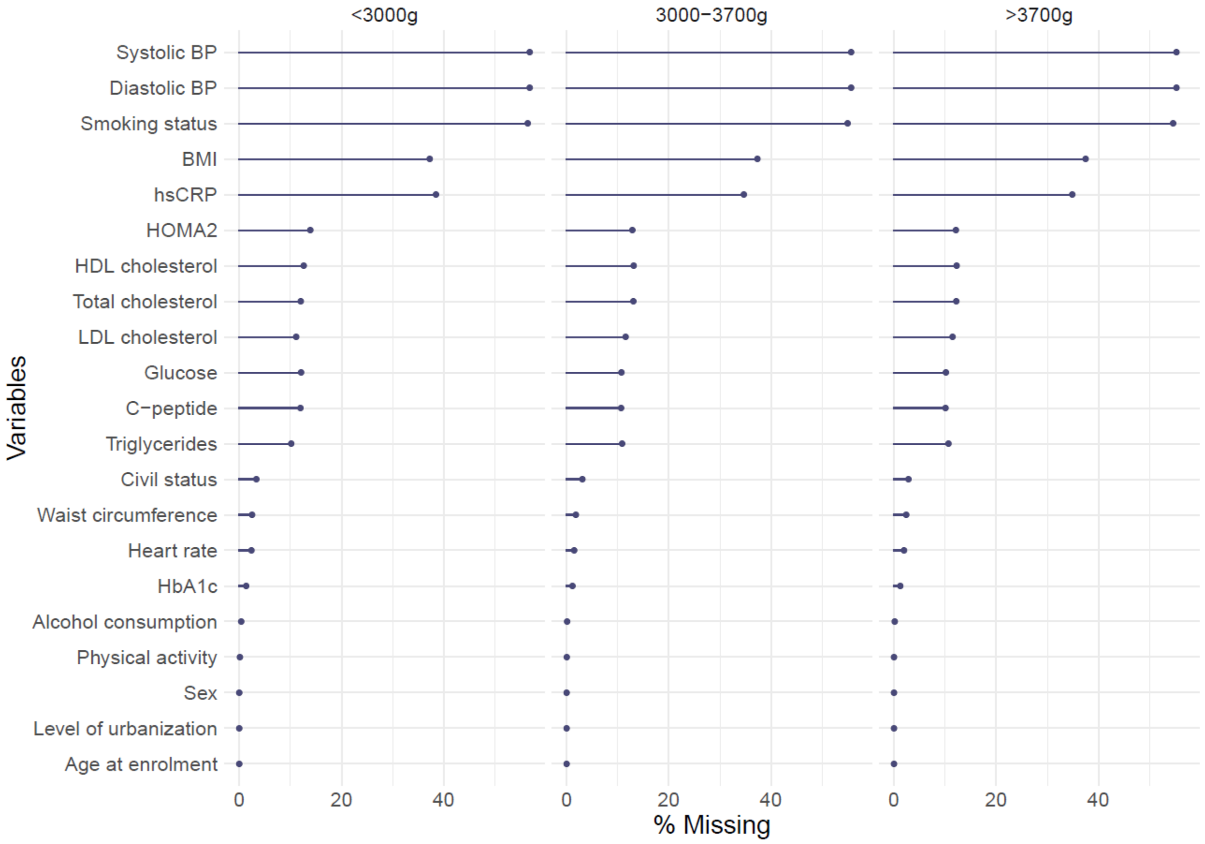

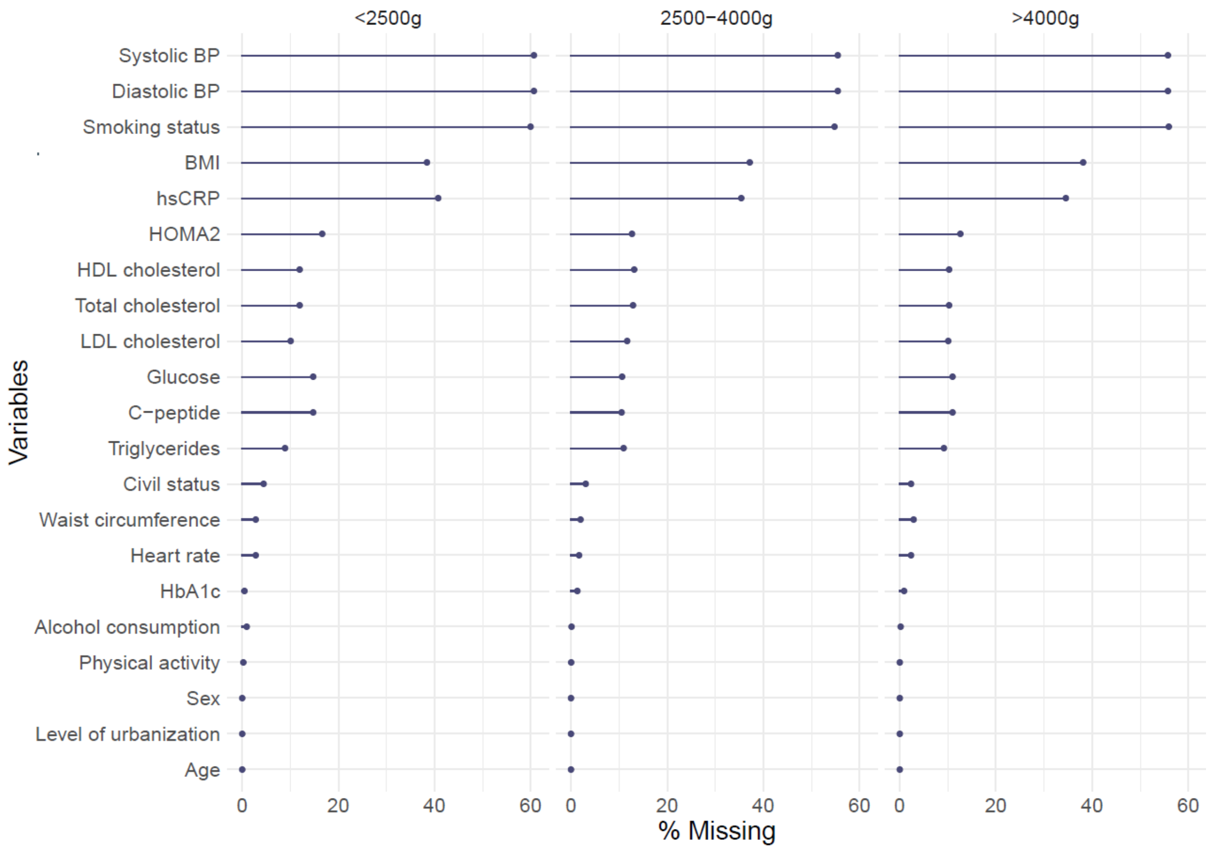
<2500g
2500−4000g
>4000g
Variables
Systolic BP
Diastolic BP
Smoking status
BMI
hsCRP
HOMA2
HDL cholesterol
Total cholesterol
LDL cholesterol
Glucose
C−peptide
Triglycerides
Civil status
Waist circumference
Heart rate
HbA1c
Alcohol consumption
Physical activity
Sex
Level of urbanization
Age
0
20
40
60
% Missing

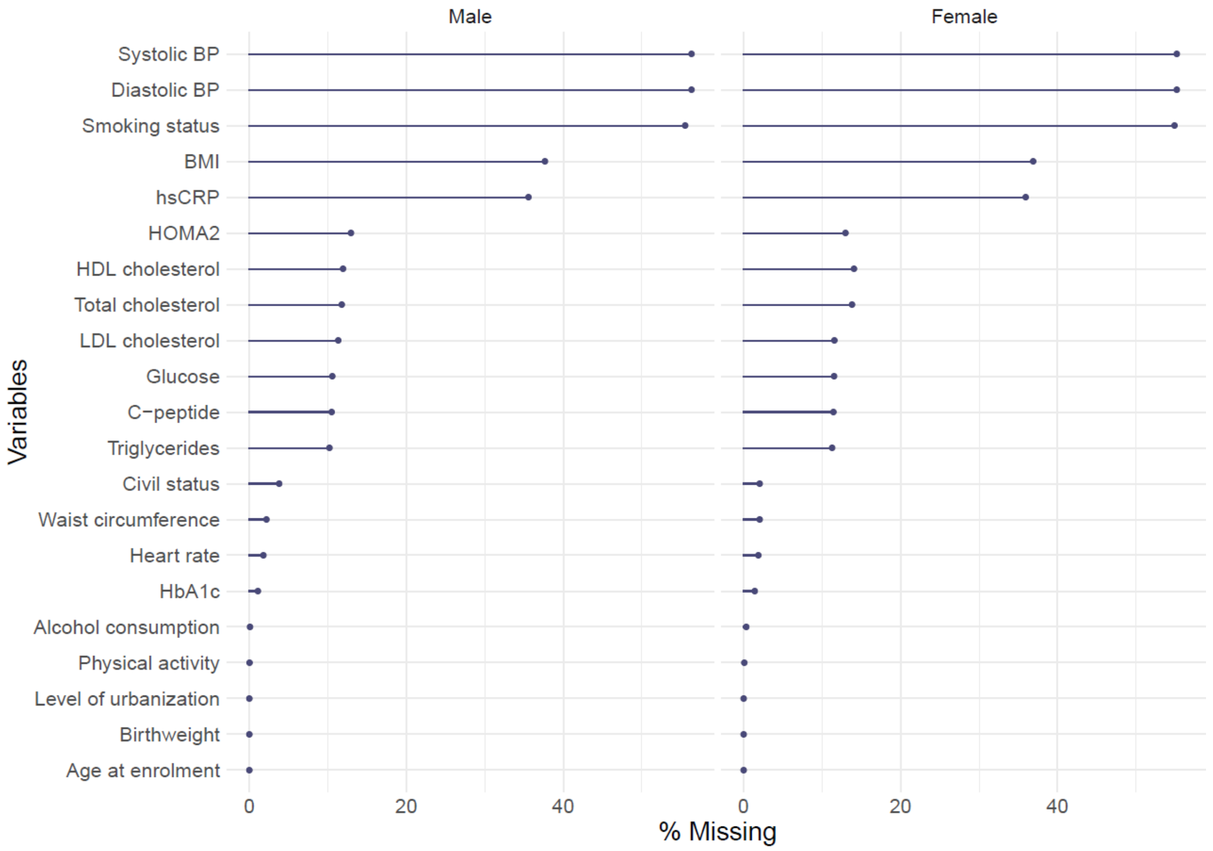
Male
Female
Variables
Systolic BP
Diastolic BP
Smoking status
BMI
hsCRP
HOMA2
HDL cholesterol
Total cholesterol
LDL cholesterol
Glucose
C−peptide
Triglycerides
Civil status
Waist circumference
Heart rate
HbA1c
Alcohol consumption
Physical activity
Level of urbanization
Birthweight
Age at enrolment
0
20
40
% Missing

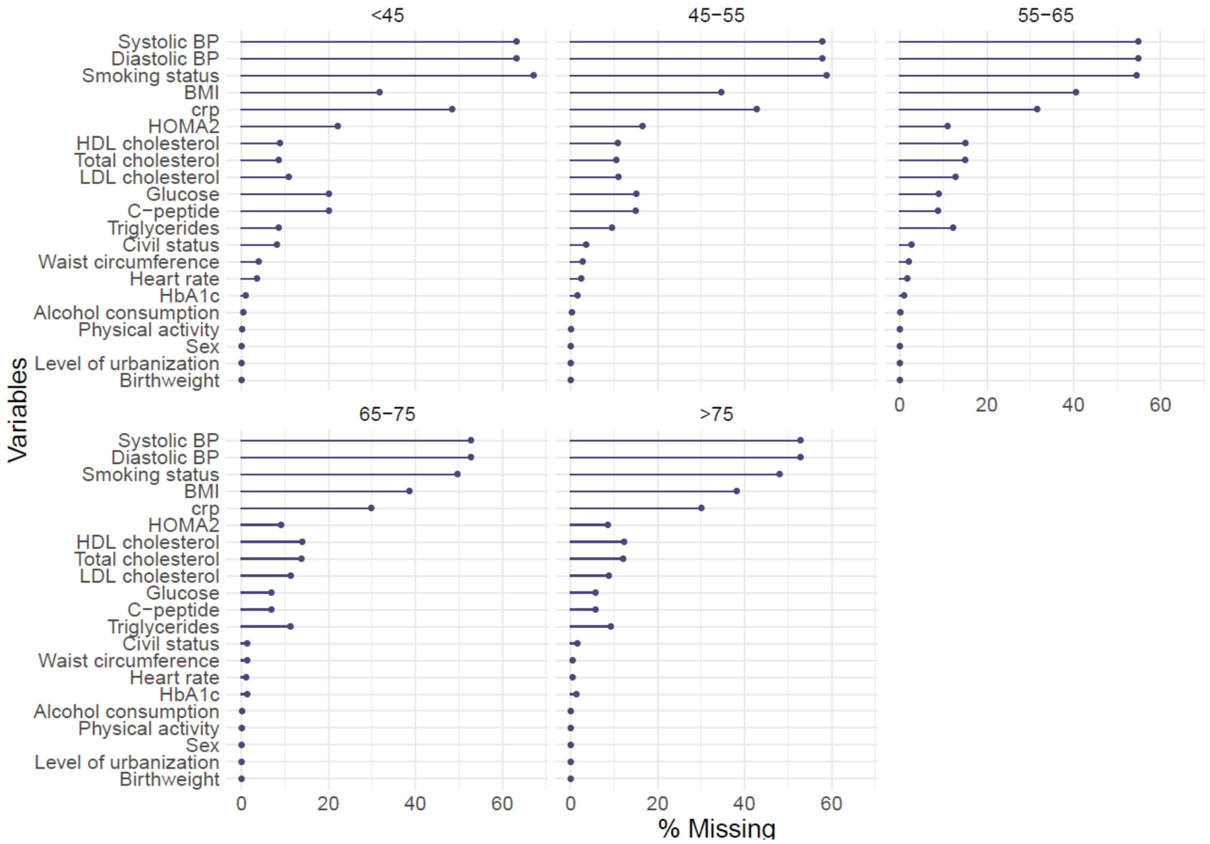

Legend: Percentage of missing data of variables used in the study. Each birthweight group is in kilograms. Abbreviations: BMI = Body Mass Index, HDL = high-density lipoprotein, LDL = low-density lipoprotein, hsCRP = high-sensitivity C-reactive protein, HOMA2 = Homeostasis Model Assessment 2. Comorbidities and medication do not have any missing data.

We used multiple imputation to create and analyse 20 imputed datasets, with the number of iterations set to 10. Incomplete variables were imputed under fully conditional specification, using the default settings of the mice 3.14.0 package[1]. The parameters of substantive interest were estimated in each imputed dataset separately and combined using Rubin's rules.

The following variables were used in the imputation model: age at enrolment, sex, alcohol consumption, physical activity, marital status, urbanization (rural-urban residence), waist circumference, BMI, family history of type 2 diabetes, HOMA2-Beta, HOMA2 insulin sensitivity, C-peptide, hsCRP, birthweight, born-at-term status, total cholesterol, LDL cholesterol, HDL cholesterol, triglycerides, smoking status, systolic blood pressure, diastolic blood pressure, HbA_{1c}, number of glucose-lowering drugs, type of glucose-lowering drugs, lipid-lowering drugs, antihypertensive medication, number of antihypertensive drugs,

calendar year at birth, and the outcomes of interest.

The following variables were imputed by the following methods:

For continuous variables, predictive mean matching was used and included weight, waist, height, BMI, HOMA2-Beta, HOMA2 insulin sensitivity, C-peptide, hsCRP, total cholesterol, HDL cholesterol, LDL cholesterol, triglycerides, systolic blood pressure, diastolic blood pressure, and HbA_{1c}. For binary data logistic regression, imputation was used and included born-at-term status. For categorical data with more than two levels, polytomous regression imputation was used and included smoking status and marital status. We combined the raw data variables of weight and height measurements into BMI before the imputation procedure. This procedure was justified by our findings that inclusion of only the raw variables for weight and height, gave rise to imprecise imputations, as outlines in a previous study[2]. Overall, the observed and imputed values had similar distributions, as shown in the density plots below.

Density plot of imputations:

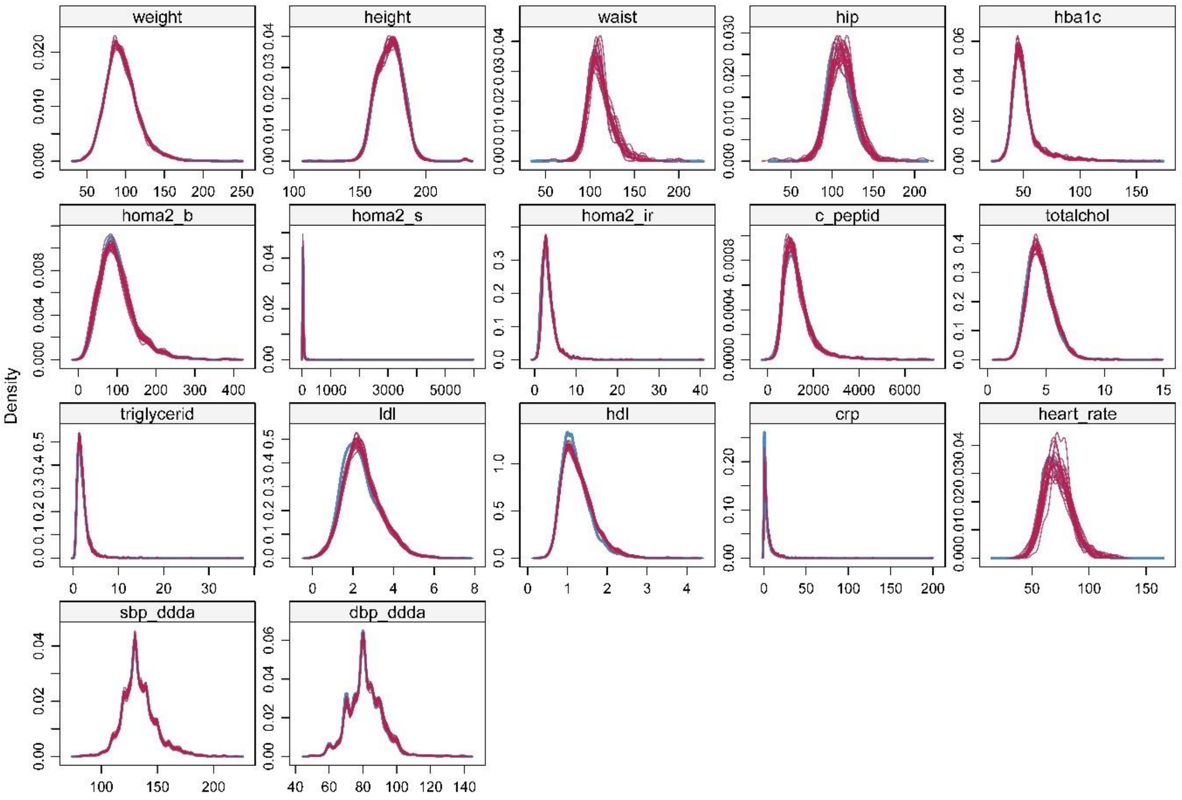

Red = Imputations, Blue = Original data.

Variables in plot:waist = waist circumference; homa2_b = HOMA2 Beta; homa2_s = HOMA2-insulin sensitivity; c_peptid = C-peptide; crp= C-reactive protein; totalchol = total cholesterol; hdl = HDL cholesterol;

sbp_ddda = systolic blood pressure; dbp_ddda = diastolic blood pressure; hba1c_nip = HbA_{1c}; ldl = LDL cholesterol; triglycerid = triglycerides.

ESM Table 1: data sources

Data sources	Description
DD2 biobank[3]	DD2 is a prospective, nationwide population-based cohort of recently diagnosed type 2 diabetes individuals, with collection of matching interview data and biological samples for a biobank at baseline. Enrolment started in November 2010 and is still ongoing.
DD2 questionnaire[4]	
The Danish Adult Diabetes Registry[5] (DDDA)	The database was established to assess quality in diabetes care on a national level in 2004. It only covers a subset of the diabetes population.
The Danish National Patient Registry[6]	Covers all inpatient (somatic) hospital contacts since 1977 and from 1995 all inpatient and outpatient hospital contacts in Denmark. Diagnostic information is coded according to the International Classification of Diseases, Tenth Revision (ICD-10) from 1994 onwards. We retrieved information from 1994 and onwards.
The Danish National Prescription Registry[7] (DNHSP)	Covers all redeemed prescriptions at Danish pharmacies since 1995.
Civil Registration System[8]	All citizens of Denmark are registered in this system by a unique Civil Personal Register number linked to registrations of birth, address, marital status, kinship, and migration among others. The Civil Personal Register number is used as linkage to other databases.
The Danish Registry of Causes of Death[9]	Death and cause of death among citizens in Denmark are recorded in this database since 1970. Both the immediately cause and the underlying cause of death are registered. Causes of death were coded according to the International Classification of Diseases, Tenth Revision (ICD-10) from 1994 onwards.
The Danish Medical Birth Register[10]	The register includes data on all birth in Denmark since 1973. It contains information of the health of pregnant women and their offspring.
National Lab Database (Laboratoriedatabasen)	The National Lab Database is comprised of detailed information of all laboratory tests carried out at Denmark's larger biochemical and immunological laboratories. It started in 2008 and complete coverage of the different regions in Denmark's starting from 2010.

ESM Table 2: Variable definitions

Variable	Source	Remarks
Biological sex	Civil Registration System	Categorical variable: male or female
Age at enrolment	Civil Registration System and DD2	Age at study enrolment in DD2<br>Continuous variable (years)<br>Categorical variable:<br><45<br>45-55<br>55-65<br>65-75<br>>75
Age at diagnosis	Civil Registration System and DD2	Defined as age as whichever of the following events came first:<br>1. First prescription for glucose-lowering drugs<br>2. First diabetes-related diagnosis in the Danish National Patient Registry<br>3. DDDA registration<br>4. First measured $HbA1_c$ ≥48 mmol/mol<br>5. DD2 enrolment<br>Continuous variable (years)
Family history of type 2 diabetes	DD2 questionnaire	Self-reported at DD2 study enrolment. Comprised of mother, father, grandparent and sibling<br>Categorical variable (number of affected relatives)
Enrolment status	DD2	Patients were enrolled either at general practitioners or hospital outpatient clinics.<br>Categorical variable
Weight at enrolment	DD2 or DDDA	If weight recorded as part of the DD2 enrolment process is available (few [not part of the DD2 core data initially]), we used that weight measure, otherwise the DDDA weight closest to DD2 enrolment, with a maximum of 2 years prior to enrolment.<br>Weights below 35 and above 300 kg were considered outliers.<br>Continuous variable (kg)
Height	DD2, DD2 2016 questionnaire or DDDA	Data on height is available from three different sources:<br>DD2 enrollment, DDDA data (repeated measures), DD2 Diabetic neuropathy questionnaire 2016 (self-reported).<br><br>Regarding DDDA data: a mean height based on all available DDDA heights were calculated for all patients 18 years or older.<br><br>We do not expect height to change over time among these adults.<br>Thus, we used the available heights in hierarchically order; height from DD2 enrolment (measured by health personal, but very few available), DD2 questionnaire survey in 2016, DDDA.

		Heights below 130 cm and above 220 were considered outliers and not included in any calculations.<br>Continuous variable (cm)
Body mass index (BMI) at enrolment	DD2, DD2 2016 questionnaire and/or DDDA	BMI were calculated using standard formula of weight/height (in meter)^2<br>BMI below 15kg/m^2 and above 70kg/m^2 were considered outliers and not included in any calculations.<br>Continuous variable (kg/m^2)
Waist circumference	DD2	Measured at DD2 enrolment.<br>Waist circumference below 50cm and above 184cm were considered outliers and not included in any calculations.<br>Continuous variable (cm)
Hip circumference	DD2	Measured at DD2 enrolment.<br>Hip circumference below 44cm and above 175cm were considered outliers and not included in any calculations.<br>Continuous variable (cm)
Waist-hip ratio	DD2	Waist and hip circumference were measured at DD2 enrolment.<br>Waist-hip ratios below 0.5 and above 1.44 were considered outliers and not included in any calculations.<br>Continuous variable (ratio)
Waist-height ratio	DD2, DD2 2016 questionnaire and/or DDDA	Waist circumference measured at DD2 enrolment.<br>Height: See above.<br>Waist-height ratio above 0.9 was considered outliers and not included in any calculations.<br>Continuous variable (ratio)
Alcohol consumption	DD2	Self-reported at enrolment. Units/week<br><br>High risk intake: >14/21 drinks per week for female/male.<br>High-risk alcohol consumption was categorized according to the Danish Health Authority's definitions as more than 21 and 14 drinks weekly for men and women, respectively in 2010 when the DD2 was initiated.<br>Categorical variable
Smoking status	DDDA	Self-reported. Collected from DDDA, using the value closest to DD2 enrolment date and a maximum 2 years prior to enrolment.<br>Categories:<br>• Never smoker<br>• Former smoker<br>• Current smoker (comprised of daily and occasionally)
Physical activity	DD2	Self-reported at DD2 enrolment: "days per week of minimum 30 minutes of moderate to vigorous activity"<br>Categorical variable (days pr week)
Marital status	Civil registration system	Collected from the Civil registration system, using the closest registration prior to DD2 enrolment date.<br>Categories:

		• Married/registered partnership.<br>• Divorced/separated.<br>• Widow/widower.<br>• Never married/registered partnership
Rural-urban residence	Statistics Denmark	Collected from Statistics Denmark, using the closest registration prior to DD2 enrolment date. Urbanization levels was categorized according to municipal level groups defined at Statistics Denmark - Municipality Groups v1:2018- - Statistics Denmark (dst.dk)<br>The five urbanization levels are based on 1) the largest city in the municipality and 2) availability of jobs (direct and indirect). Municipalities with an availability of ≥ 200,000 are capital municipalities. Municipalities with <200,000 availability in jobs and ≥ 100,000 citizens are defined as large city municipalities. Provincial municipalities are defined as <200,000 job availability and between 30,000-100,000 citizens. Surrounding Area municipalities are <30,000 citizens and 40,000-200,000 job availability. Rural municipalities are the rest.
Systolic blood pressure	DDDA	Collected from DDDA, using the value closest to DD2 enrolment date and maximum 2 year prior to enrolment.<br>Systolic blood pressure below 90mmHg and above 260mmHg were considered outliers and not included in any calculations.<br>Continuous variable (mmHg)
Diastolic blood pressure	DDDA	Collected from DDDA, using the value closest to DD2 enrolment date and maximum 2 year prior to enrolment.<br>Diastolic blood pressure below 30mmHg and above 140mmHg were considered outliers and not included in any calculations.<br>Continuous variable (mmHg)
Total cholesterol	DDDA,<br>National Lab database (NPU-codes: NPU01566, NPU18412)	Collected from DDDA and the National Lab database, using the value closet to enrolment date and maximum 2 year prior to enrolment.<br>Total cholesterol above 9.87mmol/L were considered outliers and not included in any calculations.<br>Continuous variable (mmol/L)
Triglycerides	DDDA,<br>National Lab database (NPU-codes: NPU04094, NPU03620)	Collected from DDDA and the National Lab database, using the value closet to enrolment date and maximum 2 year prior to enrolment.<br>Triglycerides above 9.30 mmol/L were considered outliers and not included in any calculations.<br>Continuous variable (mmol/L)

High Density Lipoprotein (HDL) cholesterol	DDDA,<br>National Lab database (NPU-codes: NPU01567, NPU10157)	Collected from DDDA and the National Lab database, using the value closet to enrolment date and maximum 2 year prior to enrolment.<br>HDL cholesterol above 3.08 mmol/L were considered outliers and not included in any calculations.<br>Continuous variable (mmol/L)
Low Density Lipoprotein (LDL) cholesterol	DDDA,<br>National Lab database (NPU-codes: NPU01568, NPU10171, DNK35308)	Collected from DDDA and the National Lab database, using the value closet to enrolment date and maximum 2 year prior to enrolment.<br>LDL cholesterol above 7 mmol/L were considered outliers and not included in any calculations.
Blood glucose	DD2 biobank	Measured at DD2 enrolment.<br>Continuous variable (mmol/L)
HbA1c	DDDA, National Lab database (NPU-codes: NPU03835, NPU27300)	Collected from DDDA and the National Lab database, using the value closet to enrolment date and maximum 2 year prior to enrolment.<br>HbA1c above 15% were considered outliers and not included in any calculations.<br>Continuous variable (mmol/mol (%))
C-peptide	DD2 biobank	Measured at DD2 enrolment.<br>Continuous variable (pmol/L).<br>C-peptide above 4672 pmol/L were considered outliers and not included in any calculations.
High sensitivity C-reactive protein (hsCRP)	DD2 biobank	High sensitivity low-grade inflammation.<br>Measured at DD2 enrolment.
GAD-Antibody	DD2 biobank	Measured at DD2 enrolment.
HOMA2<br>- Insulin sensitivity<br>- Beta-cell function	DD2 biobank	We used the revised homeostatic assessment model version 2 (HOMA2) to estimate insulin sensitivity (HOMA2S) and the beta-cell function (HOMA2B) based on fasting C-peptide and plasma glucose values, based on the DD2 biobank[11].<br>HOMA2 Sensitivity above 512 and HOMA2 Beta above 320 were considered outliers and not included in any calculations.
Antihypertensive drug usage	DNHSP	Up to one year prior to enrolment. Classes and ATC codes:<br>**ACE inhibitors or angiotensin II receptor antagonists:** C09A, C09B, C09C, C09D, C10BX04, C10BX06, C10BX07, C10BX11, C10BX12, C10BX13, C10BX14, C10BX15, C10BX10<br>**Calcium channel antagonists:** C08, C09BB. C09DB, C09DX01, C09DX03, C09XA53, C09XA54, C07FB, C09BX01, C09BX03, C10BX07, C10BX09, C10BX11, C10BX14<br>**Low-ceiling diuretics:** C03A, C03B, C03EA, C07D, C09BA, C09DA, C09XA52, C09XA54, C08G, C07B, C09DX01, C09DX03, C09BX03<br>**Potassium-sparing diuretics:** C03D, C03E

		**Beta-blockers:** C07<br>**Alpha-blockers:** C02CA04, C04CA03<br>**Central adrenerg inhibition:** C02AC05, C02AB<br>**Renin-inhibitors:** C09XA, C09DX02
Number of antihypertensive medications	DNHSP	Up to one year prior to enrolment.<br>One or more hits in one group counts as one agent: Thiazides, potassium-sparing diuretics, beta-blockers, Calcium channel antagonists, ACE inhibitors or ATII antagonists, Renin inhibitors, Alpha-blockers (Doxazosin), Central adrenerg inhibition (Monoxidin, methyldopa).
Lipid lowering drug usage	DNHSP	Up to one year prior to enrolment.<br>ATC: C10, A10BH51
GLP-1-analouge or SGLT2 inhibitor usage	DNSHP	GLP1-analogue or SGLT2 inhibitor use Yes/No<br>ATC:<br>**GLP-1 analogues:** A10BX04, A10BX07, A10BX10, A10BX13, A10BX14, A10BJ, A10AE54, A10AE56<br>**SGLT2-inhibitors:** A10BX09, A10BX11, A10BX12, A10BD15, A10BD16, ADBD19, A10BD20, ADBD21, A10BK, A10BD23, A10BD24
Insulin usage	DNHSP	Insulin use yes/no. Up to one-year prior enrolment.<br>ATC: A10A
Other glucose-lowering medication	DNSHP	other glucose-lowering (metformin, **DPP-4 inhibitors, SU and Meglitinides, Thiazolidinediones and Alfa-glucosidase inhibitors)** yes/No (A10)<br>Classes and ATC codes:<br>**Metformin:** A10BA, A10BD02, A10BD03, A10BD05, A10BD07, A10BD08, A10BD10, A10BD11, A10BD13, A10BD14, A10BD15, A10BD16, A10BD17, A10BD18, A10BD20, A10BD22<br>**DPP-4 inhibitors:** A10BH, A10BD07, A10BD08, A10BD09, A10BD10, A10BD11, A10BD12, A10BD13, A10BD18, A10BD19, A10BD21, A10BD22<br>**SU and meglitinides:** A10BB, A10BD04, A10BD02, A10BD06, A10BD01, A10BC01. A10BX02, A10BX03, A10BX08, A10BD14<br>**Thiazolidinediones:** A10BG, A10BD03, A10BD04, A10BD05, A10BD06, A10BD09, A10BD12<br>**Alfa-glucosidase inhibitors:** A10BF, A10BD17
Number of different glucose-lowering medications	DNHSP	Up to one year prior to enrolment.<br>One hit or more hits in one group counts as one agent: Sulfonylureas+ Meglitinides, Metformin, alpha-glucosidase inhibitors, Glitazones, DD4-inhibitors, GLP-1

		analogues, SGLT-2 inhibitors, Fast-acting insulin, Long-acting insulin
P-HbA1c	National Lab database	NPU/DNK codes:<br>NPU03835: in % (9626)<br>NPU27300: mmol/mol (235,110)<br><br>Transformation to mmol/mol has been made by the following formula:<br>HbA1c(%)*10.93 – 23.5
P-C-reactive protein	National Lab database	NPU/DNK codes:<br>NPU19748: mg/l (162,122)
P-Triglyceride	National Lab database	NPU/DNK codes:<br>NPU04094: mmol/l (128,078)<br>NPU03620: mmol/l (27,075)
P-total cholesterol	National Lab database	NPU/DNK codes:<br>NPU01566: mmol/l (153,539)<br>NPU18412: mmol/l (2112)
P-HDL cholesterol	National Lab database	NPU/DNK codes:<br>NPU01567: mmol/l (151,645)<br>NPU10157: mmol/l (2105)
P-LDL cholesterol	National Lab database	NPU/DNK codes:<br>NPU01568: mmol/l (112,728)<br>NPU10171: mmol/l (9917)<br>DNK35308: mmol/l (24,899)
Polygenic risk scores (PRS)<br>A total of 10162 individuals enrolled in the DD2 cohort 2010-2023 were genotyped using the Global Screening Array-24 v2.0 or v3.0 chip (Illumina, San Diego, CA, USA). Genotypes were called with the Illumina GenCall algorithm. Quality control excluded individuals based on sex mismatches, relatedness closer than 3rd degree, >5% missing genotypes, heterozygosity outliers, and non-European ethnicity.<br>PRS for type 2 diabetes[12], birthweight[13], chronic kidney disease[14]		

ESM Table 3: Outcome definitions

Variables	Definition	Source
Incident chronic kidney disease	Two outpatient measures of eGFR <60 ml /min per 1.73m^2 or two urine albumin-creatinine ratios >30 mg/g ≥ 90 days apart < 365 days apart	Outpatient lab measurements from the National Lab Database.<br><br>All measurements not taken during hospital admission or non-scheduled hospital visit.<br><br>Date of second measure is used as date of CKD outcome
Drop in KDIGO category	Two eGFR <60ml/min per 1.73m^2 ≥90 days apart and ≤365 days apart and the second measurement has to be a least one drop in KDIGO category (i.e., minimum from G3a → G3b or more) and at least a 25% drop in eGFR from first measurement	Outpatient lab measurements from the National Lab Database.<br><br>All measurements not taken during hospital admission or non-scheduled hospital visit.<br><br>Date of second measure is used as date of CKD outcome

ESM Table 4: Baseline characteristics at enrolment according to conventional birthweight categories

**Variable**	**<3000g (n=1,554)**	**3000-3700g (n=3,049)**	**>3700g (n=1,379)**	**Total (n=5,982)**
Sex				
Female	764 (49.2)	1,217 (39.9)	424 (30.8)	2,405 (40.2)
Age at DD2 enrolment (years)				
Median [IQR]	56.6 [48.2, 65.2]	60.8 [51.5, 68.0]	62.9 [53.0, 69.6]	60.3 [50.7, 67.8]
<45	243 (15.6)	349 (11.4)	145 (10.5)	737 (12.3)
45–55	459 (29.5)	686 (22.5)	267 (19.4)	1,412 (23.60)
55–65	457 (29.4)	932 (30.6)	369 (26.8)	1,758 (29.4)
65–75	315 (20.3)	849 (27.8)	455 (33.0)	1,619 (27.1)
>75	80 (5.1)	233 (7.6)	143 (10.4)	456 (7.6)
Family history of type 2 diabetes (n)				
0	774 (49.8)	1,485 (48.7)	641 (46.5)	2,900 (48.5)
1	456 (29.3)	944 (31.0)	443 (32.1)	1,843 (30.8)
2	240 (15.4)	455 (14.9)	210 (15.2)	905 (15.1)
+3	84 (5.4)	165 (5.4)	85 (6.2)	334 (5.6)
Marital status				
Married/partnership	870 (57.9)	1,748 (59.2)	816 (60.9)	3,434 (59.2)
Divorced/separated	276 (18.4)	512 (17.3)	206 (15.4)	994 (17.1)
Widow/widower	85 (5.7)	219 (7.4)	127 (9.5)	431 (7.4)
Non-married/no-registered partnership	271 (18.0)	476 (16.1)	191 (14.3)	938 (16.2)
Missing	52	94	39	185
Rual-urban residence				
Capital municipalities	305 (19.6)	589 (19.3)	248 (18.0)	1,142 (19.1)
Large city municipalities	337 (21.7)	679 (22.3)	299 (21.7)	1,315 (22.0)
Provincial municipalities	382 (24.6)	760 (24.9)	342 (24.8)	1,484 (24.8)
Surrounding area municipalities	280 (18.0)	525 (17.2)	257 (18.6)	1,062 (17.8)
Rural area municipalities	250 (16.1)	496 (16.3)	233 (16.9)	979 (16.4)
Born-at-term status				
Preterm	635 (40.9)	174 (5.7)	71 (5.2)	880 (14.7)
BMI (kg/m^2)				
Median [IQR]	31.1 [27.6, 35.1]	31.2 [27.8, 35.9]	31.6 [28.1, 36.2]	31.3 [27.8, 35.8]
<25	120 (12.3)	206 (10.8)	82 (9.5)	408 (10.9)
25–30	287 (29.4)	587 (30.7)	258 (29.9)	1,132 (30.2)
30–35	318 (32.6)	569 (29.8)	246 (28.5)	1,133 (30.2)
35–40	159 (16.3)	295 (15.4)	161 (18.7)	615 (16.4)
>40	91 (9.3)	254 (13.3)	115 (13.3)	460 (12.3)
Missing	579	1138	517	2234
Waist circumference (cm)				
Median [IQR]	107 [101, 116]	108 [101, 117]	110 [103, 118]	108 [102, 117]
Missing	39	55	33	127
Alcohol consumption				
>14/21 units per week (m/f)	66 (4.3)	189 (6.2)	86 (6.3)	341 (5.7)

Smoking status				
Never	342 (50.5)	637 (46.4)	277 (44.2)	1,256 (46.90)
Former	196 (29.0)	505 (36.8)	214 (34.2)	915 (34.2)
Current	139 (20.5)	231 (16.8)	135 (21.6)	505 (18.9)
Missing	877	1676	753	3306
Physical activity (days per week)				
0	193 (12.4)	412 (13.5)	188 (13.6)	793 (13.3)
1–2	356 (22.9)	642 (21.1)	273 (19.8)	1,271 (21.3)
3–4	361 (23.3)	724 (23.8)	311 (22.6)	1,396 (23.3)
5–6	276 (17.8)	517 (17.0)	223 (16.2)	1,016 (17.0)
7	366 (23.6)	753 (24.7)	384 (27.8)	1,503 (25.1)
Systolic BP (mmHg)				
Median [IQR]	131 [125, 141]	132 [125, 141]	130 [124, 142]	131 [125, 141]
	883	1697	762	3342
Diastolic BP (mmHg)				
Median [IQR]	80 [75.5, 88.0]	80 [75, 88]	80 [75, 87]	80 [75, 88]
	883	1697	762	3342
Total cholesterol (mmol/l)				
Median [IQR]	4.3 [3.7, 5.1]	4.3 [3.7, 5.1]	4.3 [3.6, 5.0]	4.3 [3.7, 5.1]
	187	398	168	753
Triglycerides (mmol/l)				
Median [IQR]	1.8 [1.3, 2.7]	1.8 [1.2, 2.6]	1.7 [1.2, 2.4]	1.8 [1.2, 2.6]
	158	331	147	636
High-density lipoprotein cholesterol (mmol/l)				
Median [IQR]	1.1 [0.9, 1.4]	1.1 [1.0, 1.4]	1.1 [0.9, 1.4]	1.1 [1.0, 1.4]
	196	400	169	765
Low-density lipoprotein cholesterol (mmol/l)				
Median [IQR]	2.2 [1.7, 2.9]	2.2 [1.7, 2.9]	2.2 [1.7, 2.9]	2.2 [1.7, 2.9]
	173	352	158	683
Blood glucose (mmol/l)				
Median [IQR]	7.3 [6.4, 8.5]	7.3 [6.4, 8.4]	7.2 [6.4, 8.5]	7.3 [6.4, 8.5]
	188	327	140	655
HbA_{1c} (mmol/mol [%])				
Median [IQR] mmol/mol	49 [44, 55]	48 [44, 55]	48 [44, 55]	48 [44, 55]
Missing	21	35	17	73
C-peptide (pmol/l)				
Median [IQR]	1,210 [920, 1,670]	1,23 [910, 1,650]	1,230 [900, 1,680]	1,220 [910, 1,660]
Missing	186	325	139	650
HOMA2-beta				
Median [IQR]	94 [69.4, 126.3]	94.7 [70.5, 124.0]	96.1 [70.6, 125.8]	94.6 [70.2, 125.1]
Missing	216	392	167	775
HOMA2-insulin sensitivity				
Median [IQR]	33.0 [24.1, 44.2]	32.5 [24.4, 45.1]	32.7 [24.1, 45.0]	32.6 [24.3, 44.8]

Missing	216	392	167	775
High-sensitivity C-reactive protein (mg/l)				
Median [IQR]	2.1 [0.9, 4.6]	2.1 [0.8, 4.7]	2.0 [0.8, 4.2]	2.1 [0.9, 4.6]
Missing	598	1057	481	2136

Legend: Continuous variables are presented in median [interquartile range]. Categorical variables presented in numbers (percentage).

ESM Table 5: Medication according to birthweight categories

Variable		<3000g (n=1,554)	3000-3700g (n=3,049)	>3700g (n=1,379)	Total (n=5,982)
Antihypertensive medication excl. loop diuretics					
	Yes	1,048 (67.4)	2,131 (69.9)	982 (71.2)	4,161 (69.6)
ACE-inhibitors/AT2					
	Yes	755 (48.6)	1,543 (50.6)	685 (49.7)	2,983 (49.9)
Number of antihypertensive medication (n)					
	0	506 (32.6)	918 (30.1)	397 (28.8)	1,821 (30.4)
	1	439 (28.3)	765 (25.1)	385 (27.9)	1,589 (26.6)
	2	317 (20.4)	747 (24.5)	312 (22.6)	1,376 (23.0)
	3+	292 (18.8)	619 (20.3)	285 (20.7)	1,196 (20.0)
Lipid-lowering medication					
	Yes	1,062 (68.3)	2,099 (68.8)	951 (69.0)	4,112 (68.7)
Glucose-lowering medication					
	No use	181 (11.7)	386 (12.7)	177 (12.8)	744 (12.4)
	oral only	1,248 (80.3)	2,452 (80.4)	1,106 (80.2)	4,806 (80.3)
	insulin+oral/insulin only	103 (6.6)	188 (6.2)	87 (6.3)	378 (6.3)
	insulin only	22 (1.4)	23 (0.8)	9 (0.7)	54 (0.9)
GLP1/SGLT2					
	Yes	201 (12.9)	353 (11.6)	134 (9.7)	688 (11.5)
Number of glucose-lowering medication (n)					
	0	181 (11.7)	386 (12.7)	177 (12.8)	744 (12.4)
	1	997 (64.2)	1,984 (65.1)	893 (64.8)	3,874 (64.8)
	2	285 (18.3)	520 (17.1)	243 (17.6)	1,048 (17.5)
	3+	91 (5.9)	159 (5.2)	66 (4.8)	316 (5.3)

Legend: Continuous variables are presented in median [interquartile range]. Categorical variables presented in numbers (percentage).

ESM Table 6: Medication according to birthweight categories

Variable	<2500g (n=427)	2500-4000g (n=4,999)	>4000g (n=556)	Total (n=5,982)
Antihypertensive medication excl. loop diuretics				
Yes	279 (65.3)	3,501 (70.0)	381 (68.5)	4,161 (69.6)
ACE-inhibitors/AT2				
Yes	204 (47.8)	2,515 (50.3)	264 (47.5)	2,983 (49.9)
Number of antihypertensive medication (n)				
0	148 (34.7)	1,498 (30.0)	175 (31.5)	1,821 (30.4)
1	123 (28.8)	1,314 (26.3)	152 (27.3)	1,589 (26.6)
2	75 (17.6)	1,175 (23.5)	126 (22.7)	1,376 (23.0)
3+	81 (19.0)	1,012 (20.2)	103 (18.5)	1,196 (20.0)
Lipid-lowering medication				
Yes	287 (67.2)	3,455 (69.1)	370 (66.6)	4,112 (68.7)
Glucose-lowering medication				
No use	46 (10.8)	630 (12.6)	68 (12.2)	NA
oral only	347 (81.3)	4,006 (80.1)	453 (81.5)	NA
insulin+oral/insulin only	26 (6.1)	319 (6.4)	33 (5.9)	NA
insulin only	8 (1.9)	44 (0.9)	<5	NA
GLP1/SGLT2				
Yes	73 (17.1)	564 (11.3)	51 (9.2)	688 (11.5)
Number of glucose-lowering medication (n)				
0	46 (10.8)	630 (12.6)	68 (12.2)	744 (12.4)
1	270 (63.2)	3,228 (64.6)	376 (67.6)	3,874 (64.8)
2	83 (19.4)	879 (17.6)	86 (15.5)	1,048 (17.5)
3+	28 (6.6)	262 (5.2)	26 (4.7)	316 (5.3)

Legend: Continuous variables are presented in median [interquartile range]. Categorical variables presented in numbers (percentage).

ESM Table 7: Incident and prior CKD according to birthweight categories

Variable	<3000g (n=1,554)	3000-3700g (n=3,049)	>3700g (n=1,379)	Total (n=5,982)
CKD				
Incident	363 (23.4)	768 (25.2)	370 (26.8)	1,501 (25.1)
Prevalent	143 (9.2)	295 (9.7)	151 (11.0)	589 (9.9)
CKD by eGFR only				
Incident	190 (12.2)	517 (17.0)	252 (18.3)	959 (16.0)
Prevalent	77 (5.0)	179 (5.9)	100 (7.3)	356 (6.0)
CKD by UACR only				
Incident	274 (17.6)	502 (16.5)	236 (17.1)	1,012 (16.9)
Prevalent	78 (5.0)	137 (4.5)	60 (4.4)	275 (4.6)
CKD: Drop-in-KDIGO category				
eGFR based only	121 (7.8)	285 (9.4)	137 (9.9)	543 (9.1)
UACR based only	63 (4.1)	142 (4.7)	45 (3.3)	250 (4.2)
Combined	166 (10.7)	369 (12.1)	167 (12.1)	702 (11.7)

Legend: Variables presented in numbers (percentage). Abbreviations: CKD = chronic kidney disease, eGFR = estimated glomerular filtration rate, UACR = urine albumin creatinine ratio

ESM Table 8: Incident and prior CKD according to conventional birthweight categories

Variable	<2500g (n= 427)	2500-4000g (n=4,999)	>4000g (n= 556)	Total (n=5,982)
CKD				
Incident	114 (26.7)	1,230 (24.6)	157 (28.2)	1,501 (25.1)
Prevalent	34 (8.0)	496 (9.9)	59 (10.6)	589 (9.9)
CKD by eGFR only				
Incident	64 (15.0)	782 (15.6)	113 (20.3)	959 (16.0)
Prevalent	12 (2.8)	305 (6.1)	39 (7.0)	356 (6.0)
CKD by UACR only				
Incident	78 (18.3)	846 (16.9)	88 (15.8)	1,012 (16.9)
Prevalent	24 (5.6)	228 (4.6)	23 (4.1)	275 (4.6)
CKD: Drop-in-category				
eGFR based only	31 (7.3)	452 (9.0)	60 (10.8)	543 (9.1)
UACR based only	19 (4.5)	211 (4.2)	20 (3.6)	250 (4.2)
Combined	47 (11.0)	581 (11.6)	74 (13.3)	702 (11.7)

Legend: Variables presented in numbers (percentage). Abbreviations: CKD = chronic kidney disease, eGFR = estimated glomerular filtration rate, UACR = urine albumin creatinine ratio

ESM Table 9: 10-year standardized risk and standardized risk difference estimates for incident CKD according to birthweight categories.

	10-year risk	10-year RD
	Non-standardized	
<3000 g	31.7 (28.9, 34.5)	-1.0 (-4.2, 2.4)

3000-3700 g	32.7 (30.7, 34.7)	ref
>3700 g	35.1 (32.1, 38.1)	2,4 (-1.0, 5.8)
<2500 g	37.1 (31.7, 42.5)	4.8 (-0.8, 10.4)
2500-4000 g	32.2 (30.6, 33.8)	ref
>4000 g	36.9 (32.2, 41.5)	4.7 (-0.2, 9.4)
Standardized for sex, calendar year at birth, age at enrolment, family history of type 2 diabetes, and born-at-term status		
<3000 g	31.2 (28.3, 34.2)	0.0 (-3.4, 3.6)
3000-3700 g	31.2 (29.2, 33.1)	ref
>3700 g	31.3 (28.7, 33.9)	0.1 (-2.9, 3.0)
<2500 g	36.0 (30.2, 42.2)	5.5 (-0.5, 11.8)
2500-4000 g	30.6 (29.1, 32.1)	ref
>4000 g	33.1 (29.1, 37.2)	2.5 (-1.6, 6.7)

Legend: Ten-year standardised and non-standardised risk and risk difference (RD) for incident CKD according to birthweight. Abbreviations: CKD = chronic kidney disease

ESM Table 10: Stepwise adjustments in cause-specific Cox proportional hazard regression of incident CKD, according to birthweight categories.

	**<3000 g**	**>3700 g**	**<2500 g**	**>4000 g**
Incident CKD				
Unadjusted	0.95 (0.84, 1.07)	1.09 (0.97, 1.24)	1.16 (0.96, 1.41)	1.18 (1.00, 1.39)
+ sex, calendar year at birth, family history of type 2 diabetes	1.08 (0.96, 1.23)	1.01 (0.89, 1.14)	1.35 (1.12, 1.64)	1.10 (0.93, 1.30)
+ age at enrolment	1.09 (0.96, 1.23)	1.00 (0.88, 1.13)	1.36 (1.12, 1.65)	1.09 (0.92, 1.28)
+ born-at-term status	1.00 (0.86, 1.16)	1.00 (0.88, 1.13)	1.23 (0.98, 1.55)	1.10 (0.93, 1.29)
+ lifestyle, marital status, rural-urban residence	1.01 (0.87, 1.16)	0.99 (0.87, 1.12)	1.21 (0.96, 1.53)	1.09 (0.92, 1.28)
+ BMI	1.04 (0.90, 1.20)	0.96 (0.85, 1.09)	1.25 (0.99, 1.58)	1.06 (0.89, 1.25)
+ number of antihypertensive and glucose-lowering medication	1.02 (0.88, 1.18)	1.00 (0.88, 1.14)	1.22 (0.97, 1.54)	1.10 (0.93, 1.30)
+ PRS for type 2 diabetes, birthweight, CKD	1.03 (0.89, 1,19)	0.99 (0.87, 1.12)	1.21 (0.96, 1.53)	1.09 (0.92, 1.29)

Legend: hazard ratios for incident CKD according to birthweight. Lifestyle includes alcohol consumption, smoking status, and physical activity. Abbreviations: CKD = chronic kidney disease, BMI = body mass index.

<u>ESM Table 11:</u> 10-year standardized risk and standardized risk difference estimates for CKD progression according to birthweight categories.

	10-year risk eGFR	10-year risk UACR	10-year risk Combined	10-year RD eGFR	10-year RD UACR	10-year RD Combined
Non-standardised						
<3000 g	6.3 (4.8, 7.8)	3.4 (2.4, 4.5)	9.4 (7.7, 11.3)	-1.7 (-3.5, 0.1)	-0.4 (-1.7, 1.0)	-1.3 (-3.5, 0.9)
3000-3700 g	8.0 (6.9, 9.2)	3.8 (2.9, 4.6)	10.7 (9.4, 12.1)	ref	ref	ref
>3700 g	8.4 (6.6, 10.3)	3.1 (2.1, 4.2)	10.6 (8.6, 12.6)	0.4 (-1.6, 2.6)	-0.7 (-2.0, 0.7)	-0.1 (-2.5, 2.2)
<2500 g	7.1 (4.3, 10.2)	5.6 (3.1, 8.5)	12.2 (8.5, 16.1)	-0.3 (-3.3, 2.8)	2.2 (-0.4, 5.1)	2.1 (-1.7, 6.1)
2500-4000 g	7.5 (6.6, 8.4)	3.4 (2.8, 4.0)	10.0 (9.0, 11.1)	ref	ref	ref
>4000 g	9.8 (6.9, 12.9)	3.3 (1.7, 5.2)	12.2 (9.0, 15.5)	2.3 (-0.6, 5.5)	0.0 (-1.8, 1.9)	2.2 (-1.2, 5.6)
Adjusted for sex, calendar year at birth, age at enrolment, family history of type 2 diabetes, and born-at-term status						
<3000 g	6.2 (4.7, 7.9)	3.0 (2.0, 4.2)	8.7 (7.0, 10.6)	-0.7 (-2.6, 1.2)	-0.7 (-2.1, 0.7)	-1.1 (-3.4, 1.2)
3000-3700 g	7.0 (6.0, 8.1)	3.8 (2.9, 4.7)	9.9 (8.6, 11.2)	ref	ref	ref
>3700 g	6.6 (5.2, 8.1)	3.0 (1.9, 4.1)	8.9 (7.2, 10.7)	-0.4 (-2.0, 1.3)	-0.8 (-2.1, 0.5)	-1.0 (-3.0, 1.1)
<2500 g	7.7 (4.5, 11.5)	5.1 (2.4, 9.2)	11.2 (7.3, 15.8)	1.1 (-2.1, 5.0)	1.8 (-1.0, 6.0)	2.1 (-1.9, 6.9)
2500-4000 g	6.5 (5.7, 7.4)	3.3 (2.7, 3.9)	9.1 (8.1, 10.1)	ref	ref	ref
>4000 g	7.8 (5.6, 10.3)	3.0 (1.5, 4.8)	10.2 (7.5, 13.1)	1.2 (-1.1, 3.8)	-0.2 (-1.8, 1.6)	1.1 (-1.7, 4.0)

Legend: Ten-year standardised and non-standardised risk and risk difference (RD) for CKD progression according to birthweight. Abbreviations: CKD = chronic kidney disease, eGFR = estimated glomerular filtration rate, UACR = urine albumin creatinine ratio

<u>ESM Table 12:</u> Stepwise adjustments in cause-specific Cox proportional hazard regression of CKD progression, according to birthweight categories.

CKD progression – Drop-in-KDIGO	<3000 g	>3700 g	<2500 g	>4000 g
**eGFR based only**				
Unadjusted	0.77 (0.58, 1.02)	1.06 (0.82, 1.38)	0.94 (0.60, 1.46)	1.33 (0.95, 1.86)
+ sex, calendar year at birth, family history of type 2 diabetes	0.90 (0.68, 1.19)	0.93 (0.71, 1.21)	1.13 (0.72, 1.76)	1.18 (0.84, 1.66)
+ age at enrolment	0.90 (0.68, 1.20)	0.93 (0.72, 1.21)	1.14 (0.73, 1.78)	1.19 (0.85, 1.67)
+ born-at-term status	0.89 (0.64, 1.23)	0.93 (0.72, 1.21)	1.19 (0.71, 2.01)	1.19 (0.84, 1.67)
+ lifestyle, marital status, rural-urban residence	0.89 (0.64, 1.24)	0.93 (0.71, 1.21)	1.12 (0.67, 1.88)	1.22 (0.86, 1.72)

+ BMI	0.92 (0.66, 1.28)	0.90 (0.69, 1.18)	1.15 (0.68, 1.94)	1.18 (0.83, 1.67)
+ number of antihypertensive and glucose-lowering medication	0.93 (0.67, 1.29)	0.94 (0.72, 1.23)	1.11 (0.66, 1.88)	1.23 (0.87, 1.75)
**UACR based only**				
Unadjusted	0.85 (0.67, 1.07)	1.00 (0.79, 1.26)	1.15 (0.81, 1.64)	1.29 (0.64, 2.61)
+ sex, calendar year at birth, family history of type 2 diabetes	0.97 (0.76, 1.23)	0.90 (0.71, 1.14)	1.33 (0.93, 1.89)	1.22 (0.60, 2.46)
+ age at enrolment	0.97 (0.76, 1.23)	0.89 (0.70, 1.13)	1.32 (0.92, 1.89)	1.24 (0.61, 2.51)
+ born-at-term status	0.87 (0.66, 1.16)	0.89 (0.71, 1.13)	1.23 (0.80, 1.89)	1.25 (0.62, 2.52)
+ lifestyle, marital status, rural-urban residence	0.87 (0.66, 1.16)	0.90 (0.71, 1.14)	1.20 (0.78, 1.84)	1.27 (0.63, 2.58)
+ BMI	0.89 (0.67, 1.18)	0.89 (0.70, 1.12)	1.22 (0.79, 1.88)	1.26 (0.62, 2.55)
+ number of antihypertensive and glucose-lowering medication	0.90 (0.67, 1.20)	0.89 (0.70, 1.13)	1.15 (0.74, 1.78)	1.27 (0.63, 2.58)
**Combined eGFR and UACR**				
Unadjusted	0.88 (0.60, 1.31)	0.83 (0.54, 1.26)	1.65 (0.97, 2.79)	0.99 (0.56, 1.76)
+ sex, calendar year at birth, family history of type 2 diabetes	0.96 (0.65, 1.43)	0.77 (0.51, 1.18)	1.75 (1.03, 2.98)	0.91 (0.51, 1.63)
+ age at enrolment	0.96 (0.65, 1.43)	0.78 (0.51, 1.19)	1.75 (1.03, 2.97)	0.91 (0.51, 1.63)
+ born-at-term status	0.80 (0.50, 1.27)	0.78 (0.51, 1.19)	1.58 (0.82, 3.05)	0.92 (0.51, 1.64)
+ lifestyle, marital status, rural-urban residence	0.81 (0.51, 1.29)	0.77 (0.50, 1.18)	1.59 (0.82, 3.07)	0.90 (0.50, 1.62)
+ BMI	0.83 (0.52, 1.32)	0.75 (0.49, 1.16)	1.63 (0.84, 3.16)	0.88 (0.49, 1.58)
+ number of antihypertensive and glucose-lowering medication	0.79 (0.49, 1.27)	0.76 (0.49, 1.16)	1.56 (0.79, 3.07)	0.91 (0.51, 1.64)

Legend: hazard ratios for CKD progression based on only eGFR, UACR or combined, according to birthweight. Lifestyle includes alcohol consumption, smoking status, and physical activity. Abbreviations: CKD = chronic kidney disease, BMI = body mass index, eGFR = estimated glomerular filtration rate, UACR = urine albumin creatinine ratio.

<u>ESM Table 13:</u> eGFR and UACR repeated measurements and mixed effects models according to birthweight categories.

	**Measurements per year**		**Linear**	**Spline 4 knots**
**eGFR**	Median (IQR)	Mean	estimate (95% CI)	
<3000 g	3.02 (2.08-4.17)	3.59	0.36 (-0.60, 1.31)	0.41 (-0.52, 1.33)
3000-3700 g	2.96 (2.06-4.11)	3.55	Ref	Ref
>3700 g	2.99 (2.13-4.14)	3.64	0.79 (-0.12, 1.70)	0.78 (-0.10, 1.67)
<2500 g	3.16 (2.32, 4.30)	3.83	-0.29 (-1.85, 1.28)	-0.28 (-1.79, 1.23)
2500-4000 g	2.97 (2.08-4.11)	3.57	Ref	Ref
>4000 g	2.96 (1.98-4.25)	3.50	0.25 (-1.00, 1.50)	0.37 (-0.85, 1.58)
Continuous birthweight (kg)	3.00 (2.09-4.13)	3.58	0.36 (-0.36, 1.08)	0.35 (-0.35, 1.05)
**UACR**			*Estimate in %*	
<3000 g	1.25 (1.04-1.63)	1.56	2.18 (-4.40, 9.21)	1.78 (-4.68, 8.68)
3000-3700 g	1.22 (1.00-1.57)	1.53	Ref	Ref
>3700 g	1.22 (1.00-1.59)	1.47	-3.73 (-9.61, 2.53)	-3.53 (-9.34, 2.65)
<2500 g	1.30 (1.06-1.76)	1.98	11.99 (0.42, 24.87)	12.59 (1.17, 25.32)
2500-4000 g	1.22 (1.00-1.57)	1.49	Ref	Ref
>4000 g	1.19 (0.97-1.55)	1.34	-7.61 (-24.96, 13.75)	-5.23 (-22.81, 16.36)
Continuous birthweight (kg)	1.23 (1.00-1.58)	1.52	-6.80 (-11.36, -1.99)	-6.58 (-11.09, -1.85)

Legend: Repeated measures of eGFR and UACR since DD2 enrolment, was used as dependent variable in mixed effects models. Fixed effects were time from DD2 enrolment until eGFR/UACR measurement, birthweight, and confounders adjusted for (sex, age at enrolment, calendar year at birth, family history of type 2 diabetes, and born-at-term status). Random effect included: time since DD2 enrolment until eGFR/UACR measurement and individual patients. UACR estimates are presented as % change per increase in one kg of birthweight, due to UACR being log transformed. Estimate for continuous birthweight is change per kg of birthweight. Abbreviations; eGFR = estimated glomerulo filtration rate, UACR = urine albumin/creatinine ratio.

<u>ESM Table 14:</u> Sub distributional hazard ratios from Fine-Gray models for incident CKD, according to birthweight.

	**<3000 g**	**>3700 g**	**<2500 g**	**>4000 g**
**Incident CKD**				
Unadjusted	0.96 (0.85, 1.09)	1.07 (0.95, 1.21)	1.19 (0.98, 1.45)	1.17 (0.99, 1.38)
+ sex, calendar year at birth, family history of type 2 diabetes	1.08 (0.95, 1.22)	0.99 (0.87, 1.12)	1.39 (1.14, 1.68)	1.09 (0.93, 1.29)
+ age at enrolment	1.08 (0.96, 1.23)	0.98 (0.87, 1.11)	1.40 (1.16, 1.70)	1.09 (0.92, 1.29)
+ born-at-term status	0.99 (0.86, 1.15)	0.99 (0.87, 1.12)	1.27 (1.01, 1.60)	1.10 (0.93, 1.30)

Legend: Sub-distributional hazard ratios for incident CKD according to birthweight. Abbreviations: CKD = chronic kidney disease, BMI = body mass index.

ESM Table 15: Cause-specific Cox proportional hazard regression of incident CKD by birthweight, excluding CKD cases diagnosed within six months before DD2 enrolment.

	**<3000 g**	**>3700 g**	**<2500 g**	**>4000 g**
	Incident CKD			
Main analysis	1.00 (0.86, 1.16)	1.00 (0.88, 1.13)	1.23 (0.98, 1.55)	1.10 (0.93, 1.29)
Excluding CKD cases within six months before enrolment	0.99 (0.86, 1.15)	1.00 (0.88, 1.14)	1.22 (0.97, 1.54)	1.11 (0.93, 1.31)

Legend: hazard ratios for incident CKD according to birthweight. CKD cases within six months of DD2 enrolment are excluded. Analyses are adjusted for sex, calendar year at birth, family history of type 2 diabetes, age at enrolment, and born-at-term status. Abbreviations: CKD = chronic kidney disease, BMI = body mass index.

ESM Table 16: Sex stratified 10-year standardized risk and standardized risk difference estimates for incident CKD according to birthweight categories.

	**10-year risk**		**10-year RD**	
	**Male**	**Female**	**Male**	**Female**
	Unadjusted			
<3100 g/<2950 g	34.3 (30.8, 37.9)	28.0 (23.9, 32.3)	2.0 (-2.3, 6.2)	-4.4 (-9.4, 0.6)
3100-3750 g/2950-3600 g	32.3 (29.6, 35.0)	32.4 (29.4, 35.5)	ref	ref
>3750 g/>3600 g	37.4 (33.6, 41.2)	32.5 (28.0, 37.0)	5.1 (0.7, 9.5)	0.1 (-5.2, 5.4)
<2500 g	39.4 (31.6, 47.2)	34.3 (26.7, 42.1)	6.4 (-1.6, 14.5)	3.3 (-4.6, 11.2)
2500-4000 g	33.0 (30.9, 35.2)	31.0 (28.6, 33.6)	ref	ref
>4000 g	39.3 (33.8, 44.8)	31.4 (23.2, 39.9)	6.3 (0.4, 12.1)	0.3 (-8.0, 8.9)
Adjusted for sex, calendar year at birth, age at enrolment, family history of type 2 diabetes, and born-at-term status				
<3100 g/<2950 g	33.7 (30.2, 37.4)	27.6 (23.1, 32.3)	3.0 (-1.3, 7.3)	-3.1 (-8.6, 2.6)
3100-3750 g/2950-3600 g	30.8 (28.3, 33.4)	30.7 (27.7, 33.7)	ref	ref
>3750 g/>3600 g	33.6 (30.3, 37.0)	29.4 (25.4, 33.7)	2.8 (-1.2, 6.7)	-1.3 (-5.8, 3.5)
<2500 g	36.6 (28.4, 45.3)	34.8 (26.5, 43.7)	5.1 (-3.4, 14.0)	5.6 (-3.1, 14.6)
2500-4000 g	31.5 (29.5, 33.5)	29.2 (26.9, 31.5)	ref	ref
>4000 g	35.6 (30.8, 40.6)	28.6 (21.3, 36.6)	4.1 (-0.9, 9.1)	-0.5 (-8.1, 7.5)

Legend: Ten-year standardised risk and risk difference (RD) for incident CKD according to birthweight stratified by sex. Abbreviations: CKD = chronic kidney disease.

ESM Table 17: Sex-stratified stepwise adjustments in cause-specific Cox proportional hazard regression of incident CKD, according to birthweight categories.

Incident CKD				
	**<3100 g**	**>3750 g**	**<2500 g**	**>4000 g**
**Male**				
Unadjusted	1.06 (0.91, 1.24)	1.21 (1.03, 1.42)	1.21 (0.93, 1.56)	1.24 (1.02, 1.51)
+ sex, calendar year at birth, family history of type 2 diabetes	1.19 (1.02, 1.40)	1.13 (0.97, 1.33)	1.38 (1.07, 1.79)	1.17 (0.96, 1.42)

+ age at enrolment	1.21 (1.03, 1.41)	1.12 (0.96, 1.31)	1.40 (1.08, 1.81)	1.16 (0.95, 1.41)
+ born-at-term status	1.13 (0.95, 1.34)	1.12 (0.96, 1.32)	1.20 (0.88, 1.66)	1.17 (0.96, 1.42)
+ lifestyle, marital status, rural-urban residence	1.14 (0.96, 1.35)	1.10 (0.94, 1.30)	1.19 (0.86, 1.63)	1.14 (0.94, 1.39)
+ BMI	1.17 (0.98, 1.40)	1.07 (0.91, 1.26)	1.20 (0.87, 1.66)	1.11 (0.91, 1.35)
+ number of antihypertensive and glucose-lowering medication	1.11 (0.93, 1.32)	1.10 (0.93, 1.29)	1.19 (0.86, 1.65)	1.14 (0.93, 1.39)
	**<2950 g**	**>3600 g**	**<2500 g**	**>4000 g**
**Female**				
Unadjusted	0.83 (0.67, 1.01)	0.99 (0.81, 1.22)	1.12 (0.84, 1.50)	1.00 (0.72, 1.38)
+ sex, calendar year at birth, family history of type 2 diabetes	0.96 (0.78, 1.18)	0.93 (0.76, 1.14)	1.32 (0.98, 1.76)	0.96 (0.69, 1.33)
+ age at enrolment	0.96 (0.78, 1.18)	0.93 (0.76, 1.14)	1.32 (0.99, 1.77)	0.95 (0.68, 1.32)
+ born-at-term status	0.85 (0.66, 1.09)	0.93 (0.76, 1.14)	1.26 (0.90, 1.77)	0.96 (0.69, 1.33)
+ lifestyle, marital status, rural-urban residence	0.88 (0.69, 1.13)	0.92 (0.75, 1.13)	1.27 (0.91, 1.78)	0.96 (0.69, 1.34)
+ BMI	0.91 (0.71, 1.17)	0.90 (0.73, 1.11)	1.33 (0.94, 1.87)	0.94 (0.67, 1.31)
+ number of antihypertensive and glucose-lowering medication	0.90 (0.70, 1.15)	0.93 (0.76, 1.14)	1.27 (0.90, 1.78)	0.97 (0.70, 1.36)

Legend: hazard ratios for incident CKD according to birthweight stratified by sex. Lifestyle includes alcohol consumption, smoking status, and physical activity. Abbreviations: CKD = chronic kidney disease, BMI = body mass index.

<u>ESM Table 18: </u>**10-year standardized risk and standardized risk difference estimates for incident CKD based on only eGFR or only UACR, according to birthweight categories.**

	**10-year risk**		**10-year RD**	
	**eGFR**	**UACR**	**eGFR**	**UACR**
		Unadjusted		
<3000 g	15.1 (13.0, 17.3)	22.0 (19.6, 24.6)	-5.3 **(**-7.9, -2.5**)**	2.4 (-0.6, 5.4)
3000-3700 g	20.3 (18.6, 22.0)	19.7 (18.0, 21.4)	ref	ref
>3700 g	22.6 (20.1, 25.2)	20.7 (18.2, 23.2)	2.2 (-0.7, 5.2)	1.0 (-2.0, 3.9)
<2500 g	18.7 (14.5, 23.3)	23.5 (18.0, 29.8)	-0.3 **(**-4.7, 4.5**)**	3.8 (-2.0, 10.2)
2500-4000 g	19.0 (17.6, 20.3)	19.8 (18.4, 21.2)	ref	ref
>4000 g	25.5 (21.3, 29.7)	18.1 (14.5, 21.7)	6.5 (2.2, 10.8)	-1.7 **(**-5.4, 2.0**)**
Adjusted for sex, calendar year at birth, age at enrolment, family history of type 2 diabetes, and born-at-term status				
<3000 g	14.7 (12.6, 17.0)	22.5 (19.7, 25.3)	-3.9 **(**-6.6, -1.2**)**	3.4 (0.1, 6.7)
3000-3700 g	18.7 (17.2, 20.2)	19.1 (17.4, 20.8)	ref	ref
>3700 g	18.8 (16.7, 21.0)	19.0 (16.6, 21.4)	0.2 (-2.2, 2.5)	-0.1 **(**-2.9, 2.8**)**
<2500 g	19.6 (15.1, 24.5)	24.4 (19.5, 29.5)	2.4 (-2.2, 7.4)	4.1 (-1.0, 9.4)
2500-4000 g	17.2 (16.0, 18.5)	20.3 (18.9, 21.7)	ref	ref
>4000 g	21.2 (18.0, 24.6)	19.8 (16.0, 23.7)	4.0 (0.6, 7.4)	-0.5 **(**-4.5, 3.6**)**

Legend: Ten-year standardised risk and risk difference (RD) for incident CKD based on only eGFR or only UACR. Abbreviations: CKD = chronic kidney disease, eGFR = estimated glomerular filtration rate, UACR = urine albumin creatinine ratio

ESM Table 19: Cause-specific Cox proportional hazard regression of incident CKD based on only eGFR or only UACR, according to birthweight categories.

	**<3000 g**	**>3700 g**	**<2500 g**	**>4000 g**
Incident CKD by eGFR only				
Unadjusted	0.71 (0.60, 0.85)	1.13 (0.97, 1.32)	0.97 (0.74, 1.27)	1.39 (1.14, 1.71)
+ sex, calendar year at birth, family history of type 2 diabetes	0.86 (0.72, 1.03)	0.99 (0.85, 1.16)	1.23 (0.94, 1.61)	1.27 (1.03, 1.55)
+ age at enrolment	0.87 (0.73, 1.04)	1.00 (0.85, 1.17)	1.26 (0.96, 1.65)	1.27 (1.04, 1.56)
+ born-at-term status	0.76 (0.61, 0.93)	1.00 (0.86, 1.18)	1.17 (0.85, 1.60)	1.28 (1.04, 1.57)
Incident CKD by UACR only				
Unadjusted	1.12 (0.96, 1.30)	1.07 (0.91, 1.26)	1.21 (0.95, 1.54)	0.98 (0.78, 1.23)
+ sex, calendar year at birth, family history of type 2 diabetes	1.24 (1.06, 1.45)	1.00 (0.85, 1.17)	1.31 (1.03, 1.67)	0.90 (0.71, 1.13)
+ age at enrolment	1.24 (1.06, 1.45)	0.99 (0.84, 1.17)	1.31 (1.03, 1.67)	0.89 (0.71, 1.12)
+ born-at-term status	1.20 (1.01, 1.44)	0.99 (0.84, 1.17)	1.22 (0.91, 1.63)	0.90 (0.71, 1.13)

Legend: hazard ratios for incident CKD according to birthweight based on only eGFR or only UACR. Abbreviations: CKD = chronic kidney disease, eGFR = estimated glomerular filtration rate, UACR = urine albumin creatinine ratio

ESM Table 20: Cause-specific Cox proportional hazard regression of incident CKD without pre-existing cardiovascular disease, according to birthweight categories.

	**<3000 g**	**>3700 g**	**<2500 g**	**>4000 g**
Unadjusted	0.97 (0.83, 1.12)	1.01 (0.86, 1.18)	1.25 (1.00, 1.58)	1.08 (0.87, 1.33)
+ sex, calendar year at birth, family history of type 2 diabetes	1.08 (0.92, 1.25)	0.95 (0.81, 1.11)	1.43 (1.14, 1.80)	1.03 (0.83, 1.28)
+ age at enrolment	1.08 (0.93, 1.26)	0.94 (0.80, 1.10)	1.43 (1.14, 1.80)	1.02 (0.82, 1.27)
+ born-at-term status	0.98 (0.82, 1.17)	0.94 (0.80, 1.10)	1.29 (0.98, 1.69)	1.03 (0.83, 1.28)

Legend: hazard ratios for incident CKD without pre-existing CVD. Abbreviations: CKD = chronic kidney disease, CVD = cardiovascular disease

ESM Table 21: Cause-specific Cox proportional hazard regression of incident CKD restricting to individuals born-at-term, according to birthweight categories.

	**<3000 g**	**>3700 g**	**<2500 g**	**>4000 g**
Unadjusted	0.85 (0.73, 0.99)	1.09 (0.96, 1.24)	1.21 (0.85, 1.72)	1.18 (0.99, 1.39)
+ sex, calendar year at birth, family history of type 2 diabetes	0.97 (0.83, 1.13)	1.00 (0.88, 1.13)	1.34 (0.94, 1.90)	1.10 (0.93, 1.30)
+ age at enrolment	0.97 (0.83, 1.14)	0.99 (0.87, 1.12)	1.35 (0.95, 1.92)	1.09 (0.92, 1.29)

Legend: hazard ratios for incident CKD restricting to individuals born-at-term. Abbreviations: CKD = chronic kidney disease

ESM Fig. 1: Flowchart of study population.

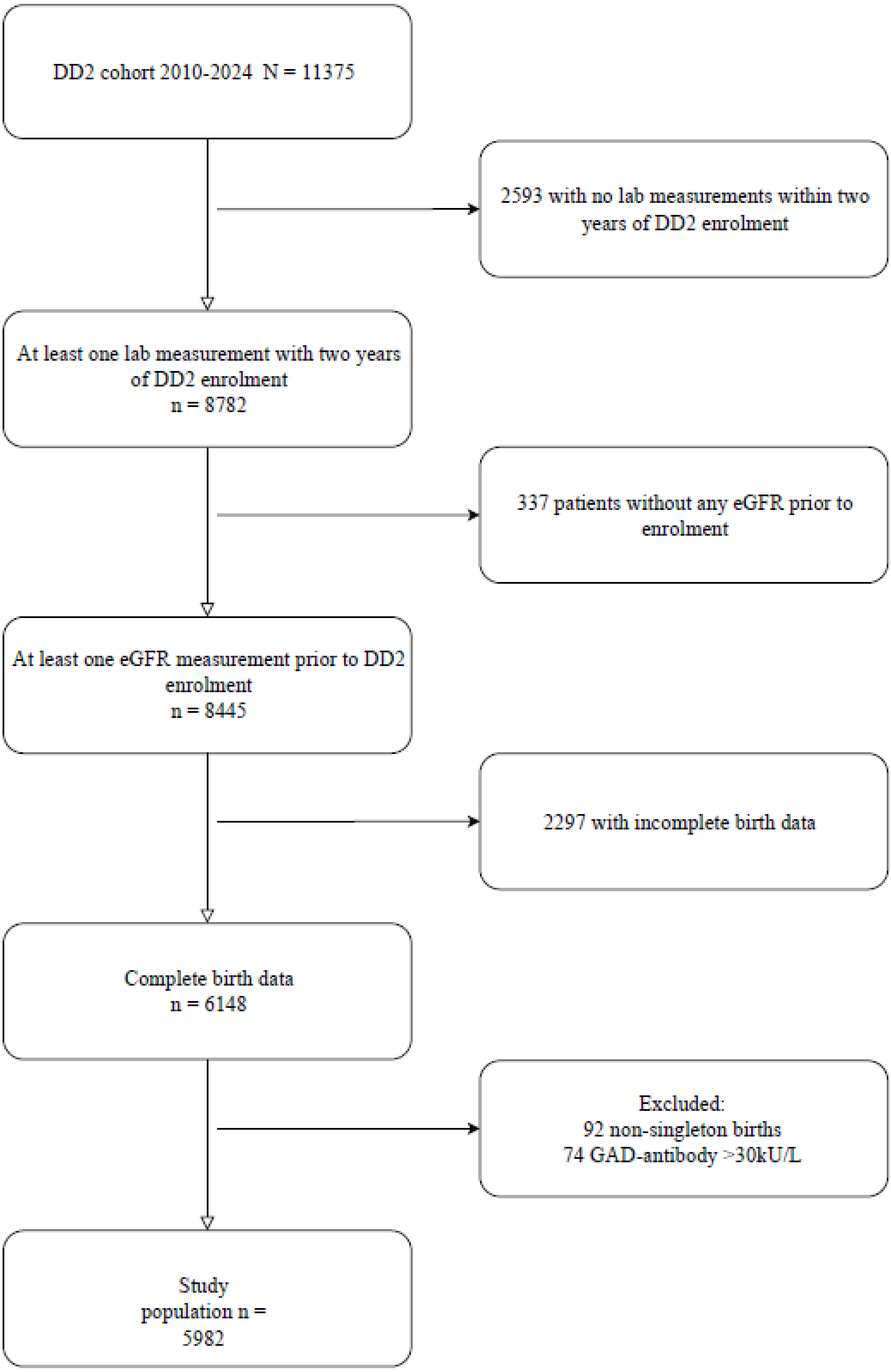

<u>ESM Fig. 2: Birthweight as a continuous exposure using restricted cubic spline regression with Cox proportional hazard regression.</u>

Spline knots were placed at fixed quantiles of the predictor's marginal distribution [15, 16]. Best models were chosen by visual inspection and lowest Akaike Information Criterion (AIC). All models were adjusted for sex, year of birth, age at enrolment, and family history of type 2 diabetes.

Default quantiles for knots placement of restricted cubic spline models:

k	Birthweights (g)
3	2750, 3400, 4000
4	2500, 3200, 3550, 4250
6	2500, 3000, 3250, 3500, 3750, 4250

Incident CKD

	**AIC**
Linear	23963
Restricted cubic spline: 3 knots	23963
Restricted cubic spline: 4 knots	23964
Restricted cubic spline: 5 knots	23966
Restricted cubic spline: 6 knots	23967

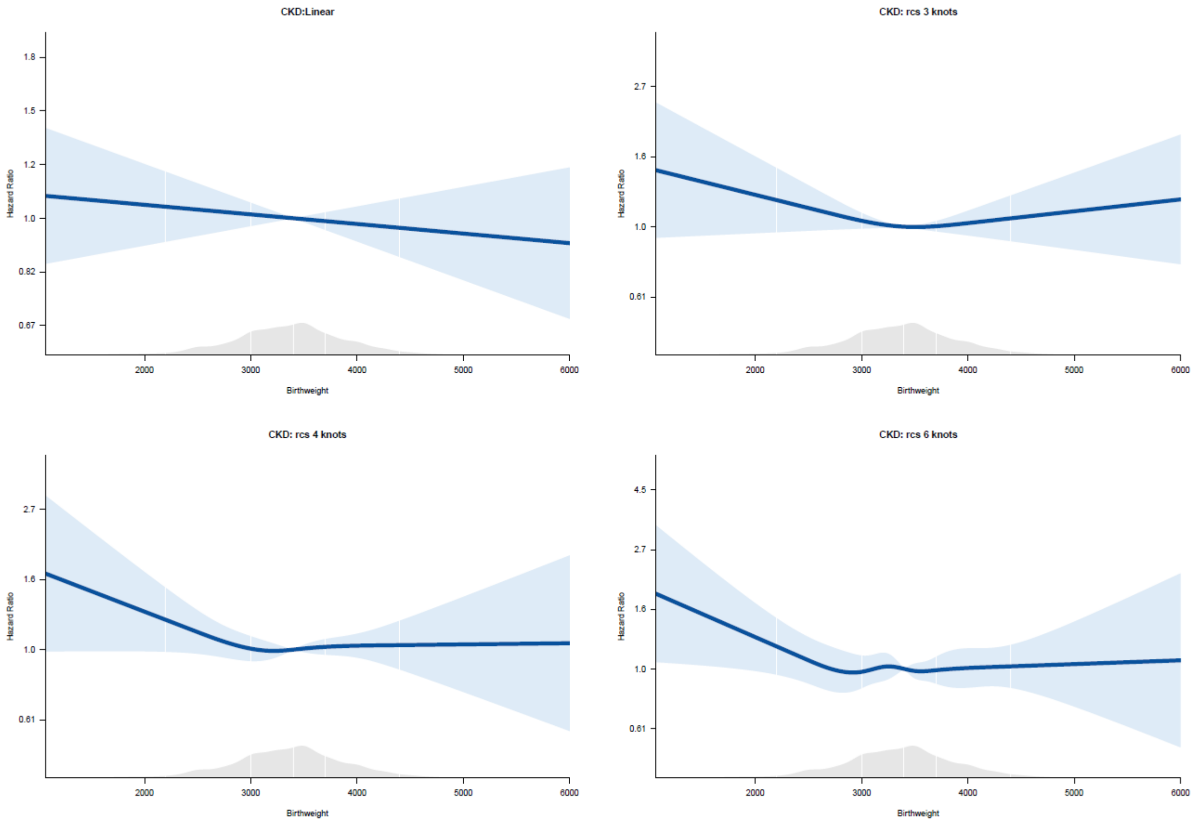

Legend: adjusted for sex, age at DD2 enrollment, calendar year of birth, family history of type 2 diabetes, and born-at-term status. Abbreviations: CKD = chronic kidney disease

CKD progression

Drop-in KDIGO category	**AIC**
eGFR only	
Linear	5053
Restricted cubic spline: 3 knots	5055
Restricted cubic spline: 4 knots	5056
Restricted cubic spline: 5 knots	5056
Restricted cubic spline: 6 knots	5056
UACR only	
Linear	2401
Restricted cubic spline: 3 knots	2403
Restricted cubic spline: 4 knots	2404
Restricted cubic spline: 5 knots	
Restricted cubic spline: 6 knots	
Combined	
Linear	6927
Restricted cubic spline: 3 knots	6929
Restricted cubic spline: 4 knots	6929
Restricted cubic spline: 5 knots	6925
Restricted cubic spline: 6 knots	6927

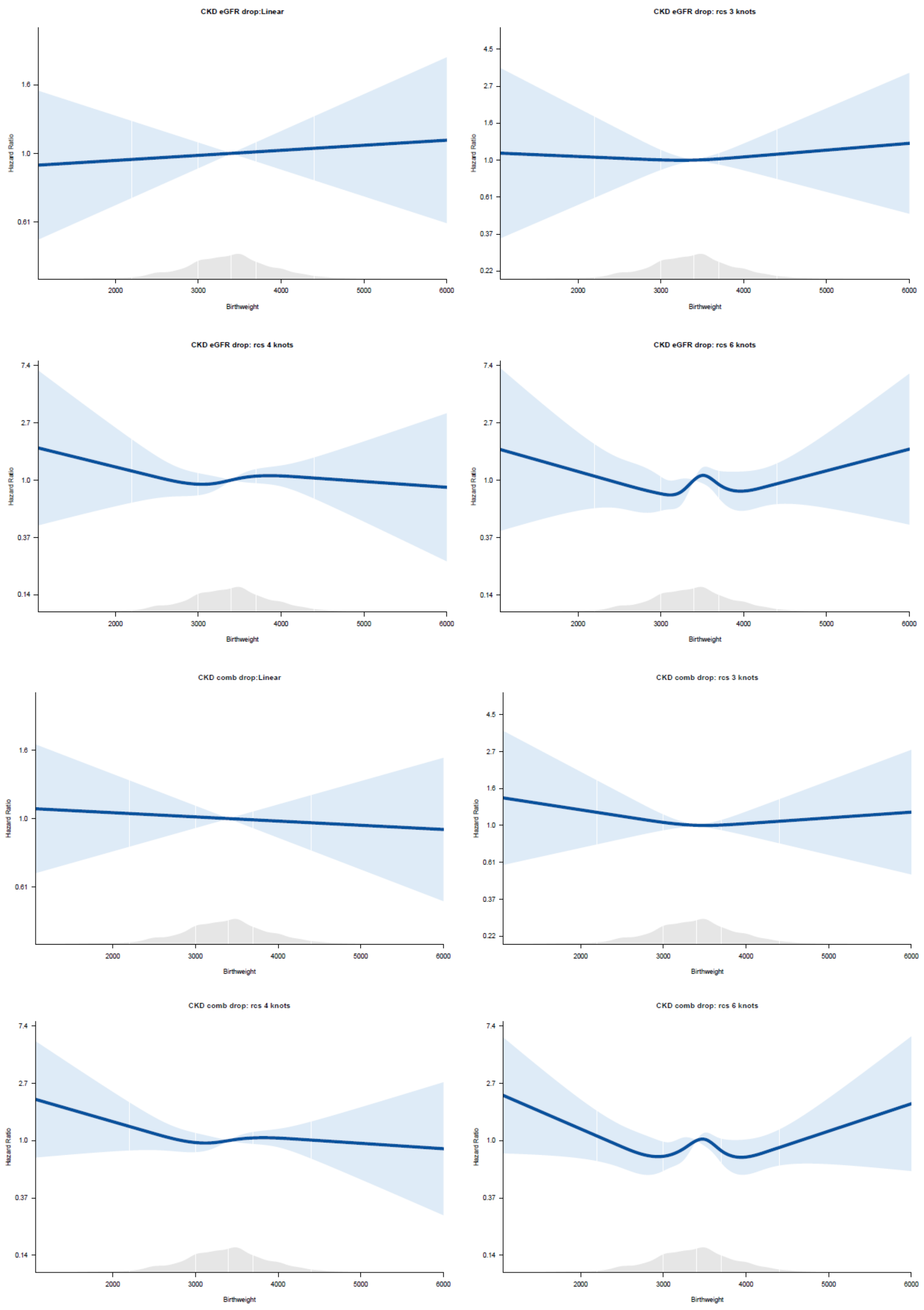

Legend: adjusted for sex, age at DD2 enrollment, calendar year of birth, family history of type 2 diabetes, and born-at-term status. Abbreviations: CKD = chronic kidney disease

Sex-stratified incident CKD

	AIC	
	Male	Female
Linear	13712	8262
Restricted cubic spline: 3 knots	13711	8264
Restricted cubic spline: 4 knots	13713	8262
Restricted cubic spline: 5 knots	13715	8262
Restricted cubic spline: 6 knots	13716	8262

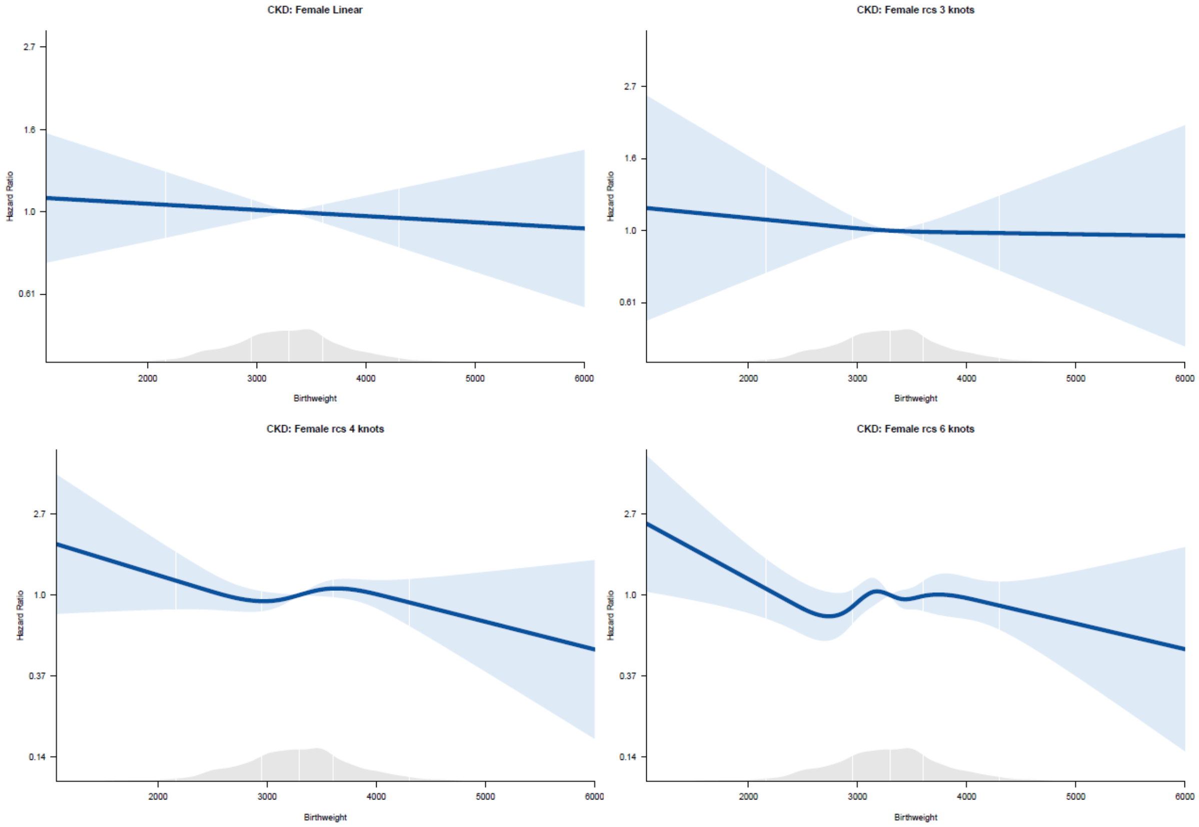

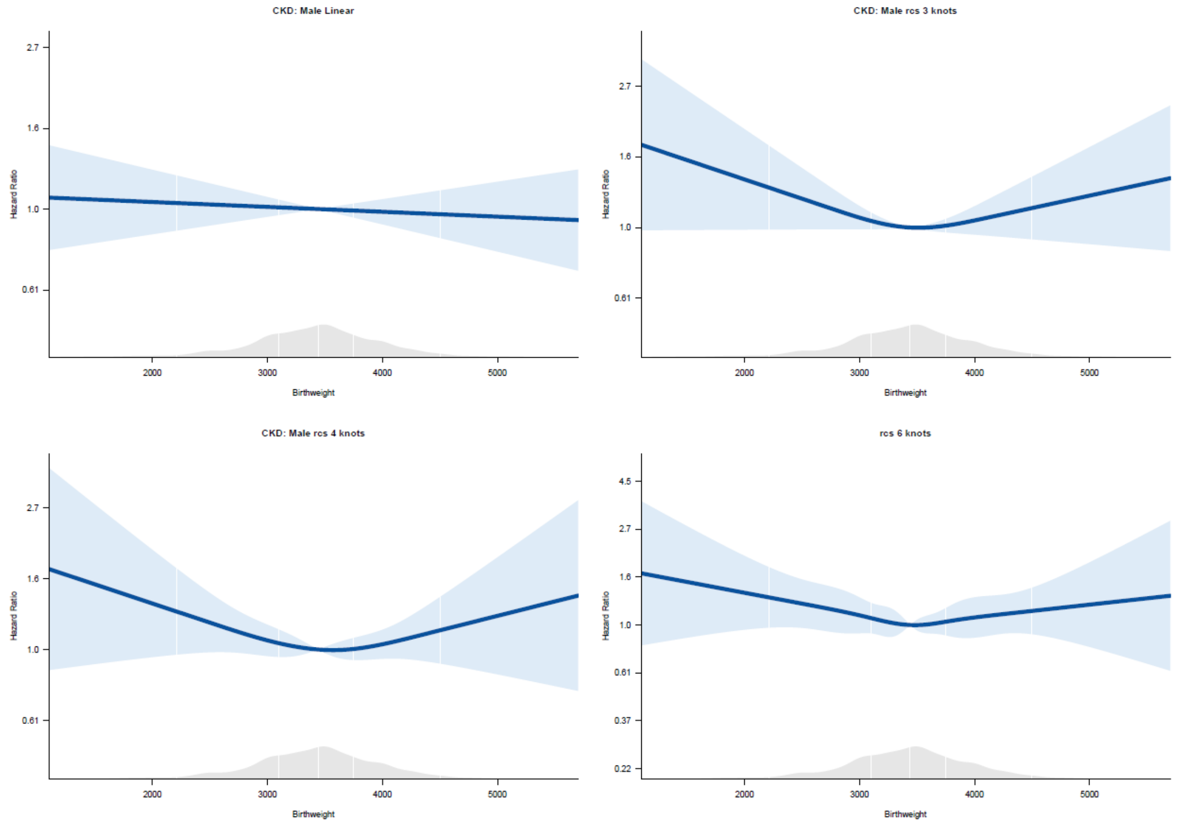

Legend: adjusted for age at DD2 enrollment, calendar year of birth, family history of type 2 diabetes, and born-at-term status. Abbreviations: CKD = chronic kidney disease

ESM Fig. 3: Sex-stratified standardized risk for incident CKD according to birthweight groups.

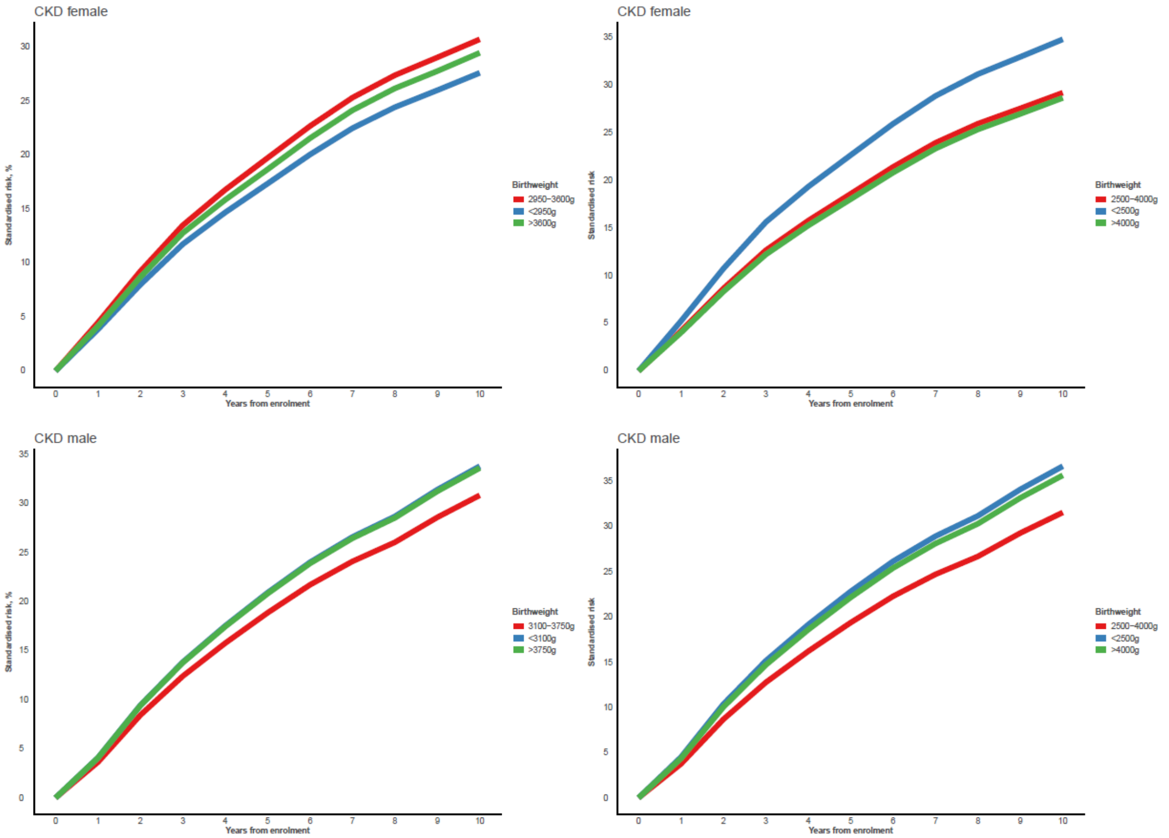

Legend: Sex-stratified standardised risk curves for incident CKD according to birthweight. Standardized to the distribution of age at DD2 enrolment, calendar year of birth, family history of type 2 diabetes, and born-at-term status. Blue lines, low birthweight group; red lines, reference group; yellow lines, high birthweight group. Abbreviations: CKD = chronic kidney disease

References

[1] van Buuren S, Groothuis-Oudshoorn K (2011) mice: Multivariate Imputation by Chained Equations in R. Journal of Statistical Software 45(3): 1 - 67. 10.18637/jss.v045.i03
[2] Hansen AL, Thomsen RW, Brøns C, et al. (2023) Birthweight is associated with clinical characteristics in people with recently diagnosed type 2 diabetes. Diabetologia. 10.1007/s00125-023-05936-1
[3] Christensen H, Nielsen JS, Sørensen KM, Melbye M, Brandslund I (2012) New national Biobank of The Danish Center for Strategic Research on Type 2 Diabetes (DD2). Clin Epidemiol 4: 37-42. 10.2147/clep.S33042
[4] Nielsen JS, Thomsen RW, Steffensen C, Christiansen JS (2012) The Danish Centre for Strategic Research in Type 2 Diabetes (DD2) study: implementation of a nationwide patient enrollment system. Clin Epidemiol 4(Suppl 1): 27-36. 10.2147/clep.S30838
[5] Jørgensen ME, Kristensen JK, Reventlov Husted G, Cerqueira C, Rossing P (2016) The Danish Adult Diabetes Registry. Clin Epidemiol 8: 429-434. 10.2147/clep.S99518
[6] Lynge E, Sandegaard JL, Rebolj M (2011) The Danish National Patient Register. Scand J Public Health 39(7 Suppl): 30-33. 10.1177/1403494811401482
[7] Pottegård A, Schmidt SAJ, Wallach-Kildemoes H, Sørensen HT, Hallas J, Schmidt M (2017) Data Resource Profile: The Danish National Prescription Registry. Int J Epidemiol 46(3): 798-798f. 10.1093/ije/dyw213
[8] Schmidt M, Pedersen L, Sørensen HT (2014) The Danish Civil Registration System as a tool in epidemiology. Eur J Epidemiol 29(8): 541-549. 10.1007/s10654-014-9930-3
[9] Helweg-Larsen K (2011) The Danish Register of Causes of Death. Scand J Public Health 39(7 Suppl): 26-29. 10.1177/1403494811399958
[10] Bliddal M, Broe A, Pottegård A, Olsen J, Langhoff-Roos J (2018) The Danish Medical Birth Register. Eur J Epidemiol 33(1): 27-36. 10.1007/s10654-018-0356-1
[11] Hill NR, Levy JC, Matthews DR (2013) Expansion of the homeostasis model assessment of β-cell function and insulin resistance to enable clinical trial outcome modeling through the interactive adjustment of physiology and treatment effects: iHOMA2. Diabetes Care 36(8): 2324-2330. 10.2337/dc12-0607
[12] Ma Y, Patil S, Zhou X, Mukherjee B, Fritsche LG (2022) ExPRSweb: An online repository with polygenic risk scores for common health-related exposures. The American Journal of Human Genetics 109(10): 1742-1760. https://doi.org/10.1016/j.ajhg.2022.09.001
[13] Privé F, Aschard H, Carmi S, et al. (2022) Portability of 245 polygenic scores when derived from the UK Biobank and applied to 9 ancestry groups from the same cohort. Am J Hum Genet 109(1): 12-23. 10.1016/j.ajhg.2021.11.008
[14] Ritchie SC, Lambert SA, Arnold M, et al. (2021) Integrative analysis of the plasma proteome and polygenic risk of cardiometabolic diseases. Nature Metabolism 3(11): 1476-1483. 10.1038/s42255-021-00478-5
[15] Stone CJ (1986) [Generalized Additive Models]: Comment. Statistical Science 1(3): 312-314
[16] Harrell FE (2016) Regression modeling strategies. Springer International Publishing, Cham, Switzerland
